# Dispersive Hydrodynamics of Soliton Condensates for the Korteweg–de Vries Equation

**DOI:** 10.1007/s00332-023-09940-y

**Published:** 2023-09-19

**Authors:** T. Congy, G. A. El, G. Roberti, A. Tovbis

**Affiliations:** 1 Department of Mathematics, Physics and Electrical Engineering, Northumbria University, Newcastle upon Tyne NE1 8ST, United Kingdom; 2 Department of Mathematics, University of Central Florida, Orlando, Florida 32816 USA

**Keywords:** Soliton gas, Kinetic equation, Integrability, Korteweg-de Vries equation, Whitham modulation equations, 35Q53, 37K40, 35L60

## Abstract

We consider large-scale dynamics of non-equilibrium dense soliton gas for the Korteweg–de Vries (KdV) equation in the special “condensate” limit. We prove that in this limit the integro-differential kinetic equation for the spectral density of states reduces to the *N*-phase KdV–Whitham modulation equations derived by Flaschka et al. (Commun Pure Appl Math 33(6):739–784, 1980) and Lax and Levermore (Commun Pure Appl Math 36(5):571–593, 1983). We consider Riemann problems for soliton condensates and construct explicit solutions of the kinetic equation describing generalized rarefaction and dispersive shock waves. We then present numerical results for “diluted” soliton condensates exhibiting rich incoherent behaviors associated with integrable turbulence.

## Introduction

Solitons represent the fundamental localized solutions of integrable nonlinear dispersive equations such as the Korteweg–de Vries (KdV), nonlinear Schrödinger (NLS), sine-Gordon, Benjamin–Ono and other equations. Along with the remarkable localization properties, solitons exhibit particle-like elastic pairwise collisions accompanied by definite phase/position shifts. A comprehensive description of solitons and their interactions is achieved within the inverse scattering transform (IST) method framework, where each soliton is characterized by a certain spectral parameter related to the soliton’s amplitude, and the phase related to its position (for the sake of definiteness, we refer here to the properties of KdV solitons). Generally, integrable equations support *N*-soliton solutions, which can be viewed as nonlinear superpositions of *N* solitons. Within the IST framework, *N*-soliton solution is characterized by a finite set of spectral and phase parameters completely determined by the initial conditions for the integrable PDE.

The particle-like properties of solitons suggest some natural questions pertaining to the realm of statistical mechanics, e.g. one can consider a *soliton gas* as an infinite ensemble of interacting solitons characterized by random spectral (amplitude) and phase distributions. The key question is to understand the emergent macroscopic dynamics (i.e. hydrodynamics or kinetics) of soliton gas given the properties of the elementary, “microscopic”, two-soliton interactions. It is clear that due to the presence of an infinite number of conserved quantities and the lack of thermalization in integrable systems the properties of soliton gases will be very different compared to the properties of classical gases whose particle interactions are non-elastic. Invoking the wave aspect of the soliton’s dual identity, soliton gas can be viewed as a prominent example of *integrable turbulence* (Zakharov 2009). The pertinent questions arising in this connection are related to the determination of the parameters of the random nonlinear wave field in the soliton gas such as probability density function, autocorrelation function, power spectrum, etc.

The IST-based phenomenological construction of a *rarefied*, or diluted, gas of KdV solitons was proposed in 1971 by Zakharov (1971) who has formulated an approximate spectral kinetic equation for such a gas based on the properties of soliton collisions: the conservation of the soliton spectrum (isospectrality) and the accumulation of phase shifts in pairwise collisions that results in the modification of an effective average soliton’s velocity in the gas. Zakharov’s spectral kinetic equation was generalized in El (2003) to the case of a dense gas using the finite gap theory and the thermodynamic, infinite-genus, limit of the KdV–Whitham modulation equations (Flaschka et al. 1980). The results of El (2003) were used in El and Kamchatnov (2005) for the formulation of a phenomenological construction of kinetic equations for dense soliton gases for integrable systems describing both unidirectional and bidirectional soliton propagation and including the focusing, defocusing and resonant NLS equations, as well as the Kaup–Boussinesq system for shallow-water waves (Congy et al. 2021). The detailed spectral theory of soliton and breather gases for the focusing NLS equation has been developed in El and Tovbis (2020).

The spectral kinetic equation for a dense soliton gas represents a nonlinear integro-differential equation describing the evolution of the *density of states* (DOS)—the density function $u(\eta ; x,t)$ in the phase space $(\eta , x) \in \Gamma ^+ \times {\mathbb {R}}$, where $\eta \in \Gamma ^+$ is the spectral parameter in the Lax pair associated with the nonlinear integrable PDE,

1.1 $$\begin{aligned}{} & {} u_t + (us)_x=0, \nonumber \\{} & {} \quad s(\eta ; x,t)=s_0(\eta ) + \int _{\Gamma ^+} G(\eta , \mu )u(\mu ; x,t)[s(\eta ; x,t)-s(\mu ; x,t)]\textrm{d}\mu .\qquad \end{aligned}$$

Here $s_0(\eta )$ is the velocity of a “free” soliton, and the integral term in the second equation represents the effective modification of the soliton velocity in the gas due to pairwise soliton collisions that are accompanied by the phase-shifts described by the kernel $G(\eta , \mu )$. Both $s_0(\eta )$ and $G(\eta , \mu )$ are system-specific. In particular, for KdV $s_0(\eta )=4\eta ^2$ and $G(\eta , \mu )= \tfrac{1}{\eta }\ln \left|\tfrac{\mu +\eta }{\mu -\eta }\right|$. The spectral support $\Gamma ^+$ of the DOS is determined by initial conditions. We note that, while $\Gamma ^+ \subset {\mathbb {R}}^+$ for the KdV equation, one can have $\Gamma ^+ \subset {\mathbb {C}}^+$ for other equations, e.g. the focusing NLS equation, see (El and Tovbis 2020). Equation (1.1) describes the DOS evolution in a *dense* soliton gas and represents a broad generalization of Zakharov’s kinetic equation for rarefied gas (Zakharov 1971). The existence, uniqueness and properties of solutions to the “equation of state” (the integral equation in (1.1) for fixed (*x*, *t*) for the focusing NLS and KdV equations were studied in Kuijlaars and Tovbis (2021).

The original spectral theory of the KdV soliton gas (El 2003) has been developed under the assumption that the spectral support $\Gamma ^+$ of the DOS is a fixed, simply-connected interval of ${\mathbb {R}}^+$; without loss of generality, one can assume $\Gamma ^+=[0,1]$. In El and Tovbis (2020), Kuijlaars and Tovbis (2021), this restriction has been removed by allowing the spectral support $\Gamma ^+$ to be a union of $N+1$ disjoint intervals $\gamma _j=[\lambda _{2j-1}, \lambda _{2j}]$, termed here *s-bands*: $\Gamma ^+= \cup _{j=0}^{N} \gamma _j$, ($\gamma _i \cap \gamma _j= \emptyset$, $i \ne j$). In this paper, we introduce a further generalization of the existing theory by allowing the endpoints $\lambda _j$ of the s-bands be functions of *x*, *t*. We show that this generalization has profound implications for soliton gas dynamics; in particular, the kinetic equation implies certain nonlinear evolution of the endpoints $\lambda _j(x,t)$ of the s-bands. We determine this evolution for a special type of soliton gases, termed in El and Tovbis (2020) *soliton condensates*. Soliton condensate represents the “densest possible” gas whose DOS is uniquely defined by a given spectral support $\Gamma ^+$. The number *N* of disjoint s-bands in $\Gamma ^+$ determines the *genus* $g=N-1$ of the soliton condensate. We show that the evolution of $\lambda _j$’s in a soliton condensate is governed by the *g*-phase-averaged KdV–Whitham modulation equations (Flaschka et al. 1980), also derived in the context of the semi-classical, zero-dispersion limit of the KdV equation (Lax and Levermore 1983).

We then consider the soliton condensate dynamics arising in the Riemann problem initiated by a rapid jump in the DOS. Our results suggest that in the condensate limit the KdV dynamics of soliton gas is almost everywhere equivalent to the (deterministic) generalized rarefaction waves (RWs) and generalized dispersive shock waves (DSWs) of the KdV equation. We prove this statement for the genus 0 case and present a strong numerical evidence for genus 1. Our results also suggest direct connection of the “deterministic KdV soliton gases” constructed in the recent paper (Girotti et al. 2021) with modulated soliton condensates.

Our work puts classical results of integrable dispersive hydrodynamics (Flaschka et al. 1980; Lax and Levermore 1983; Gurevich and Pitaevskii 1974) in a broader context of the soliton gas theory. Namely, we show that the KdV–Whitham modulation equations describe the emergent hydrodynamic motion of a special soliton gas—a condensate—resulting from the accumulated effect of “microscopic” two-soliton interactions. This new interpretation of the Whitham equations is particularly pertinent in the context of generalized hydrodynamics, the emergent hydrodynamics of quantum and classical many-body systems (Doyon 2020). The direct connection between the kinetic theory of KdV soliton gas and generalized hydrodynamics was established recently in (see also Bettelheim 2020 where the Whitham equations for the defocusing NLS equation were shown to arise in the semi-classical limit of the generalized hydrodynamics of the quantum Lieb–Liniger model).

Our work also paves the way to a major extension of the existing dispersive hydrodynamic theory by including the random aspect of soliton gases. To this end, we consider “diluted” soliton condensates whose DOS has the same spectral distribution as in genuine condensates but allows for a wider spacing between solitons giving rise to rich incoherent dynamics associated with “integrable turbulence” (Zakharov 2009). In particular, we show numerically that evolution of initial discontinuities in diluted soliton condensates results in the development of incoherent oscillating rarefaction and dispersive shock waves.

An important aspect of our work is the numerical modeling of soliton condensates using *n*-soliton KdV solutions with large *n*, configured according to the condensate DOS. The challenges of the numerical implementation of standard *n*-soliton formulae for sufficiently large *n* due to rapid accumulation of roundoff errors are known very well. Here we use the efficient algorithm developed in Huang (1992), which relies on the Darboux transformation. We improve this algorithm following the recent methodology developed in Gelash and Agafontsev (2018) for the focusing NLS equation with the implementation of high-precision arithmetic routine. Our numerical simulations show excellent agreement with analytical predictions for the solutions of soliton condensate Riemann problems and provide a strong support to the basic conjecture about the connection of KdV soliton condensates with finite-gap potentials.

It should be noted that soliton condensates have been recently studied for the focusing NLS equation, where they represent incoherent wave fields exhibiting distinct statistical properties. In particular, it was shown in Gelash et al. (2019) that the so-called bound state soliton condensate dynamics underlies the long-term behavior of spontaneous modulational instability, the fundamental physical phenomenon that gives rise to the statistically stationary integrable turbulence (Agafontsev and Zakharov 2015; Agafontsev et al. 2021).

The paper is organized as follows: in Sect. 2, we present a brief outline of the spectral theory of soliton gas for the KdV equation and introduce the notion of soliton condensate for the simplest genus 0 case. In Sect. 3, following Kuijlaars and Tovbis (2021), we generalize the spectral definition of soliton condensate to an arbitrary genus case and prove the main Theorem 3.2 connecting spectral dynamics of non-uniform soliton condensates with multiphase Whitham modulation theory (Flaschka et al. 1980) describing slow deformations of the spectrum of periodic and quasiperiodic KdV solutions. Section 3 is concerned with properties of KdV solutions corresponding to the condensate spectral DOS, i.e. the soliton condensate realizations. We formulate Conjecture 4.1 that any realization of an equilibrium soliton condensate almost surely coincides with a finite-gap potential defined on the condensate’s hyperelliptic spectral curve. This proposition is proved for genus 0 condensates, and a strong numerical evidence is provided for genus 1 and 2. In Sect. 5, we construct solutions to Riemann problems for the soliton gas kinetic equation subject to discontinuous condensate initial data. These solutions describe evolution of generalized rarefaction and dispersive shock waves. In Sect. 6, we present numerical simulations of the Riemann problem for the KdV soliton condensates and compare them with analytical solutions from Sect. 5. Finally, in Sect. 7 we consider basic properties of “diluted” condensates having a scaled condensate DOS and exhibiting rich incoherent behaviors. In particular, we present numerical solutions to Riemann problems for such diluted condensates. Appendix A contains details of the numerical implementation of dense soliton gases. In Appendix B, we present results of the numerical realization of the genus 2 soliton condensate and its comparison with two-phase solution of the KdV equation.

## Spectral Theory of KdV Soliton Gas: Summary of Results

Here we present an outline of the spectral theory of KdV soliton gas developed in El (2003, 2021). We consider the KdV equation in the form

2.1 $$\begin{aligned} \varphi _t +6\varphi \varphi _x+\varphi _{xxx} = 0. \end{aligned}$$

The inverse scattering theory associates soliton of the KdV equation (2.1) with a point $z=z_1=-\eta _1^2$, $\eta _1>0$ of the discrete spectrum of the Lax operator

2.2 $$\begin{aligned} {\mathcal {L}}= -\partial _{xx}^2 - \varphi (x,t), \end{aligned}$$

with sufficiently rapidly decaying potential $\varphi (x,t)$: $\varphi (x,t) \rightarrow 0$ as $|x| \rightarrow \infty$. The corresponding KdV soliton solution is given by:

2.3 $$\begin{aligned} \varphi _\textrm{s}(x,t;\eta _1) = 2 \eta _1^2 \hbox {sech}^2 [\eta _1(x-4\eta _1^2 t - x_1^0)], \end{aligned}$$

where the soliton amplitude $a_1= 2\eta _1^2$, the speed $s_1= 4\eta _1^2$, and $x_1^0$ is the initial position or “phase”. Along with the simplest single-soliton solution (2.3), the KdV equation supports *n*-soliton solutions $\varphi _n(x,t)$ characterized by *n* discrete spectral parameters $0< \eta _1< \eta _2< \dots <\eta _n$ and the set of initial positions $\{x_i^0 | i=1, \dots , n\}$ associated with the phases of the so-called norming constants (Novikov et al. 1984). It is also known that *n*-soliton solutions can be realized as special limits of more general *n*-gap solutions, whose Lax spectrum ${\mathcal {S}}_n$ consists of *n* finite and one semi-infinite bands separated by *n* gaps (Novikov et al. 1984; Belokolos et al. 1994),

2.4 $$\begin{aligned} z \in {\mathcal {S}}_n=[\zeta _1, \zeta _2] \cup [\zeta _3, \zeta _4] \cup \dots \cup [\zeta _{2n+1}, \infty ). \end{aligned}$$

The *n*-gap solution of the KdV equation (2.1) represents a multiphase quasiperiodic function

2.5 $$\begin{aligned} \begin{aligned}&\varphi (x,t)=F_n(\theta _1, \theta _2, \dots , \theta _n), \quad \theta _j=k_jx-\omega _j t + \theta _j^0, \\&F_n(\dots , \theta _j+ 2 \pi , \dots ) = F_n(\dots , \theta _j, \dots ), \end{aligned} \end{aligned}$$

where $k_j$ and $\omega _j$ are the wavenumber and frequency associated with the *j*-th phase $\theta _j$, and $\theta _j^0$ are the initial phases. Details on the explicit representation of the solution (2.5) in terms of Riemann theta-functions can be found in classical papers and monographs on finite-gap theory, see (Matveev 2008; Belokolos et al. 1994) and references therein.

The *n*-phase (*n*-gap) KdV solution (2.5) is parametrized by $2n+1$ endpoints $\{\zeta _j \}_{j=1}^{ 2n+1}$ of the spectral bands. The nonlinear dispersion relations (NDRs) for finite gap potentials can be represented in the general form, see (Flaschka et al. 1980) for the concrete expressions,

2.6 $$\begin{aligned} k_j=K_j(\zeta _1, \dots , \zeta _{2n+1}), \quad \omega _j=\Omega _j(\zeta _1, \dots , \zeta _{2n+1}), \quad j=1, \dots , n, \end{aligned}$$

and connect the wavenumber-frequency set $\{k_j, \omega _j\}_{j=1}^n$ of (2.5) with the spectral set ${\mathcal {S}}_n$ (2.4). These are complemented by the relation $\langle {\varphi }\rangle =\Phi (\zeta _1, \dots , \zeta _{2n+1})$, where $\langle \varphi \rangle = \int F_n \textrm{d}\theta _1 \dots \textrm{d}\theta _n$ is the mean obtained by averaging of $F_n$ over the phase *n*-torus ${\mathbb {T}}^n=[0, 2\pi ) \times \dots \times [0, 2 \pi )$, assuming respective non-commensurability of $k_j$’s and $\omega _j$’s and, consequently, ergodicity of the KdV flow on the torus.

The *n*-soliton limit of an *n*-gap solution is achieved by collapsing all the finite bands $[\zeta _{2j-1}, \zeta _{2j}]$ into double points corresponding to the soliton discrete spectral values,

2.7 $$\begin{aligned} \zeta _{2j} -\zeta _{2j-1} \rightarrow 0, \ \ \zeta _{2j}, \zeta _{2j-1} \rightarrow - \eta _j^2, \ \ j=1, \dots ,n. \end{aligned}$$

It was proposed in El (2003) that the special infinite-soliton limit of the spectral *n*-gap KdV solutions, termed the thermodynamic limit, provides spectral description the KdV soliton gas. The thermodynamic limit is achieved by assuming a special band-gap distribution (scaling) of the spectral set ${\mathcal {S}}_n$ for $n \rightarrow \infty$ on a fixed interval $[\zeta _{1}, \zeta _{2n+1}]$ (e.g. $[-1, 0]$). Specifically, we set the spectral bands to be exponentially narrow compared to the gaps so that ${\mathcal {S}}_n$ is asymptotically characterized by two smooth positive functions on some fixed interval $\Gamma ^+ \subset {\mathbb {R}}^+$: the density $\phi (\eta )$ of the lattice points $\eta _j \in \Gamma ^+$ defining the band centers via $-\eta _j^2=(\zeta _{2j}+\zeta _{2j-1})/2$, and the scaled logarithmic bandwidth distribution $\tau (\eta )$ defined for $n \rightarrow \infty$ by

2.8 $$\begin{aligned} \eta _j-\eta _{j+1} \sim \frac{1}{n \phi (\eta _j)}, \quad \tau (\eta _j) \sim -\frac{1}{n} \ln (\zeta _{2j}-\zeta _{2j-1}), \end{aligned}$$

where $\phi (\eta )$, $\tau (\eta )$ are ${\mathcal {O}}(1)$. The spectral scaling (2.8) was originally introduced by Venakides (1989) in the context of the continuum limit of theta functions.

We complement the spectral distributions (2.8) with the uniform distribution of the initial phase vector ${\varvec{\theta }}^{0}$ on the torus ${\mathbb {T}}^n$ and say that the resulting random finite gap solution $\varphi (x,t)$ approximates *soliton gas* as $n \rightarrow \infty$. An important consequence of this definition of soliton gas is ergodicity, implying that spatial averages of the KdV field in a soliton gas are equivalent to the ensemble averages, i.e. the averages over ${\mathbb {T}}^n$ in the thermodynamic limit $n \rightarrow \infty$. We shall use the notation $\langle F[\varphi ] \rangle$ for ensemble averages and $\overline{F[\varphi ]}$ for spatial averages.

From now on, we shall refer to $\eta >0$ as the spectral parameter and $\Gamma ^+$–the spectral support. The DOS $u(\eta )$ of a spatially homogeneous (equilibrium) soliton gas is phenomenologically introduced in such a way that $u(\eta _0)\textrm{d}\eta \textrm{d}x$ gives the number of solitons with the spectral parameter $\eta \in [\eta _0, \eta _0 + \textrm{d} \eta ]$ contained in the portion of soliton gas over a macroscopic (i.e. containing sufficiently many solitons) spatial interval $x \in [x_0, x_0 + \textrm{d} x] \subset {\mathbb {R}}$ for any $x_0$ (the individual solitons can be counted by cutting out the relevant portion of the gas and letting them separate with time). The corresponding spectral flux density $v(\eta )$ represents the temporal counterpart of the DOS, i.e. ${|v(\eta _0)| \textrm{d} \eta }$ is the number of solitons with the spectral parameter $\eta \in [\eta _0, \eta _0 + \textrm{d} \eta ]$ crossing any given point $x=x_0$ per unit interval of time; note that, even if the velocity of an “isolated” KdV soliton is always positive, the flux density $v(\eta )$ in the KdV soliton gas can be negative, see e.g. Remark 2.1. These definitions are physically suggestive in the context of rarefied soliton gas where solitons are identifiable as individual localized wave structures. The general mathematical definitions of $u(\eta )$ and $v(\eta )$ applicable to dense soliton gases are introduced by applying the thermodynamic limit to the finite-gap NDRs (2.6), leading to the integral equations (El 2003, 2021):

2.9 $$\begin{aligned}{} & {} \int _{\Gamma ^+} \ln \left|\frac{\mu +\eta }{\mu -\eta }\right| u(\mu )\textrm{d}\mu +u(\eta ){\sigma }(\eta )= \eta , \end{aligned}$$

2.10 $$\begin{aligned}{} & {} \int _{\Gamma ^+} \ln \left|\frac{\mu +\eta }{\mu -\eta }\right| v(\mu )\textrm{d}\mu +v(\eta ){\sigma }(\eta )= 4 \eta ^3 , \end{aligned}$$

for all $\eta \in \Gamma ^+$. Here the *spectral scaling function* $\sigma : \Gamma ^+ \rightarrow [0,\infty )$ is a continuous non-negative function that encodes the Lax spectrum of soliton gas via $\sigma (\eta )=\phi (\eta )/\tau (\eta )$. Equations (2.9), (2.10) represent the soliton gas NDRs.

Eliminating $\sigma (\eta ) \ne 0$ from the NDRs (2.9), (2.10) yields the *equation of state* for KdV soliton gas:

2.11 $$\begin{aligned} s(\eta ) = 4\eta ^2 + \frac{1}{\eta } \int _{\Gamma ^+} \ln \left| \frac{\eta +\mu }{\eta -\mu } \right| u(\mu ) [s(\eta ) - s(\mu )] \textrm{d}\mu , \end{aligned}$$

where $s(\eta )= v(\eta )/u(\eta )$ can be interpreted as the velocity of a *tracer soliton* in the gas. It was shown in El (2003) that for a weakly non-uniform (non-equilibrium) soliton gas, for which $u(\eta )\equiv u(\eta ;x,t)$, $s(\eta ) \equiv s(\eta ; x, t)$, the DOS satisfies the continuity equation

2.12 $$\begin{aligned} u_t + (us)_x=0, \end{aligned}$$

so that $s(\eta ; x, t)$ acquires the natural meaning of the transport velocity in the soliton gas. Equations (2.11), (2.12) form the spectral kinetic equation for soliton gas. One should note that the typical scales of spatiotemporal variations in the kinetic equation (2.12) are much larger than in the KdV equation (2.1), i.e. the kinetic equation describes macroscopic evolution, or hydrodynamics, of soliton gases.

Let the spectral support $\Gamma ^+$ be fixed. Then, differentiating equation (2.9) with respect to *t*, equation (2.10) with respect to *x*, and using the continuity equation (2.12), we obtain the evolution equation for the spectral scaling function

2.13 $$\begin{aligned} \sigma _t + s \sigma _x = 0, \end{aligned}$$

which shows that $\sigma (\eta ;x,t)$ plays the role of the Riemann invariant for the spectral kinetic equation.

Finally, the ensemble averages of the conserved densities of the KdV wave field in the soliton gas (the Kruskal integrals) are evaluated in terms of the DOS as $\langle {{{\mathcal {P}}}}_n[\varphi ] \rangle = C_n \int _{\Gamma ^+} \eta ^{2n-1} u(\eta )\textrm{d} \eta$, where $\mathcal{P}_n[\varphi ]$, $n=1,2, \dots$ are conserved quantities of the KdV equation and $C_n$ constants (El 2003, 2021) (see also Tovbis and Wang (2022) for rigorous derivation in the NLS context). In particular, for the two first moments we have, on dropping the *x*, *t*-dependence (El 2003, 2021),

2.14 $$\begin{aligned} \langle \varphi \rangle = 4\int _{\Gamma ^+} \eta \, u(\eta ) \textrm{d}\eta , \quad \langle \varphi ^2 \rangle = \frac{16}{3} \int _{\Gamma ^+} \eta ^3 \, u(\eta ) \textrm{d}\eta . \end{aligned}$$

We note that in the original works on KdV soliton gas it was assumed (explicitly or implicitly) that the spectral support $\Gamma ^+$ of the KdV soliton gas is a fixed, simply connected interval (without loss of generality one can assume that in this case $\Gamma ^+=[0,1]$). In what follows we will be considering a more general configuration where $\Gamma ^+$ represents a union of $N+1$ disjoint intervals, $N=0,1, \dots$. In addition, we will allow the endpoints of these intervals to be functions of *x*, *t*.

A special kind of soliton gas, termed *soliton condensate*, is realized spectrally by letting $\sigma \rightarrow 0$ in the NDRs (2.9), (2.10). This limit was first considered in El and Tovbis (2020) for the soliton gas in the focusing NLS equation and then in Kuijlaars and Tovbis (2021) for KdV. Loosely speaking, soliton condensate can be viewed as the “densest possible” gas (for a given spectral support $\Gamma ^+$) whose properties are fully determined by the interaction (integral) terms in the NDRs (2.9), (2.10).

For the KdV equation, setting $\sigma = 0$ and, considering the simplest case $\Gamma ^+=[0,1]$ in (2.9), (2.10), we obtain the soliton condensate NDRs (El 2021):

2.15 $$\begin{aligned} \int _0^1 \ln \left|\frac{\mu +\eta }{\mu -\eta }\right| u(\mu )\textrm{d}\mu = \eta , \quad \int _0^1 \ln \left|\frac{\mu +\eta }{\mu -\eta }\right| v(\mu )\textrm{d}\mu = 4 \eta ^3. \end{aligned}$$

These are solved by

2.16 $$\begin{aligned} u(\eta )= \frac{\eta }{\pi \sqrt{1-\eta ^2}},\quad v(\eta )= \frac{6\eta (2\eta ^2-1)}{\pi \sqrt{1-\eta ^2}}, \end{aligned}$$

as verified by direct substitution (it is advantageous to first differentiate equations (2.15) with respect to $\eta$). The formula (2.16) for $u(\eta )$ is sometimes called the Weyl distribution, following the terminology from the semiclassical theory of linear differential operators (Ivrii 2016; Lax and Levermore 1983).

### Remark 2.1

The meaning of the zero $\eta _0= 1/{\sqrt{2}}$ of $v(\eta )$ is that all the tracer solitons with the spectral parameter $\eta >\eta _0$ move to the right, whereas all the tracer solitons with $\eta <\eta _0$ move in the opposite direction, while the tracer soliton with $\eta =\eta _0$ is stationary. The somewhat counter-intuitive “backflow” phenomenon (we remind that KdV solitons considered in isolation move to the right) has been observed in the numerical simulations of the KdV soliton gas (Pelinovsky and Shurgalina 2017) and can be readily understood from the phase shift formula of two interacting solitons, where the larger soliton gets a kick forward upon the interaction, while the smaller soliton is pushed back. As a matter of fact, the KdV soliton backflow is general and can be observed for a broad range of sufficiently dense gases (see Fig. 16 in Sect. 7.1 for the numerical illustration).

## Soliton Condensates and Their Modulations

We now consider the general case of the soliton gas NDRs (2.9), (2.10) by letting the support $\Gamma ^+$ of $u(\eta ), v(\eta )$ to be a union of disjoint intervals $\gamma _k \subset {{\mathbb {R}}}^+$, $k=0,1, \dots ,$*N* where $\gamma _0=[0,\lambda _1]$ and $\gamma _j=[\lambda _{2j},\lambda _{2j+1}]$, $j=1, \dots , N$, i.e.

3.1 $$\begin{aligned} \Gamma ^+ = [0, \lambda _1] \cup [\lambda _2, \lambda _3] \cup \dots \cup [\lambda _{2N}, \lambda _{2N+1}]. \end{aligned}$$

We shall call the intervals $\gamma _k$ the *s-bands*, and the soliton gas spectrally supported on $\Gamma ^+$ (3.1)— the *genus N soliton gas*. Correspondingly, we refer to the intervals $c_j=(\lambda _{2j-1}, \lambda _{2j})$ separating the s-bands as to s-gaps. Note that the s-bands and s-gaps are different from the original bands and gaps in the spectrum ${\mathcal {S}}_n$ of finite-gap potential (cf. (2.4)) as they emerge *after* the passage to the thermodynamic limit: loosely speaking, one can view the s-bands as a continuum limit of the “thermodynamic band clusters”, each representing an isolated dense subset of ${\mathcal {S}}_\infty$ consisting of the collapsing original bands. The existence and uniqueness of solutions $u(\eta )$, $v(\eta )$ for (2.9), (2.10), respectively, as well as the fact that $u(\eta )\ge 0$ on $\Gamma ^+$ with some mild constraints, were established in Kuijlaars and Tovbis (2021). Our goal here is to find explicit expressions for *u*, *v* for the genus *N* soliton condensate, that is, solutions of (2.9), (2.10) for the particular case ${\sigma }\equiv 0$ on $\Gamma ^+$.

Denote by $\Gamma ^-$ the symmetric image of $\Gamma ^+$ with respect to the origin, i.e., $\Gamma ^-=-\Gamma ^+$. If we take the odd continuation of *u*, *v* to $\Gamma ^-$ (preserving the same notations), we observe that equations (2.9), (2.10) become

3.2 $$\begin{aligned}{} & {} -\int _\Gamma \ln |\mu -\eta | u(\mu )\textrm{d}\mu +u(\eta ){\sigma }(\eta )= \eta , \end{aligned}$$

3.3 $$\begin{aligned}{} & {} -\int _\Gamma \ln |\mu -\eta | v(\mu )\textrm{d}\mu +v(\eta ){\sigma }(\eta )= 4 \eta ^3, \end{aligned}$$

where $\Gamma :=\Gamma ^+\cup \Gamma ^-$, for all $\eta \in \Gamma ^+$. In fact, if we symmetrically extend ${\sigma }(\eta )$ from $\Gamma ^+$ to $\Gamma$, equations (3.2), (3.3) should be valid on $\Gamma$ since every term in these equations is odd. The expressions (2.14) for the first two moments (ensemble averages) of the KdV wave field in the soliton gas become

3.4 $$\begin{aligned} \langle \varphi \rangle = 2 \int _\Gamma \eta \, u(\eta ) \textrm{d}\eta ,\ \qquad \langle \varphi ^2 \rangle = \frac{8}{3} \int _{\Gamma }\eta ^3 \, u(\eta ) \textrm{d}\eta . \end{aligned}$$

We now consider soliton condensate of genus *N* by setting ${\sigma }\equiv 0$ in (3.2), (3.3). Then, differentiating in $\eta$ we obtain

3.5 $$\begin{aligned} H[u]=\frac{1}{\pi }, \quad H[v]=\frac{12\eta ^2}{\pi } \quad \text {on }\Gamma , \end{aligned}$$

where *H* denotes the finite Hilbert transform (FHT) on $\Gamma$, see for example (Tricomi 1951; Okada and Elliot 1991),

3.6 $$\begin{aligned} H [f] (\xi )= \frac{1}{\pi } \int _{\Gamma } \frac{ f(y)\textrm{d}y}{y-\xi }. \end{aligned}$$

Equations (3.5) are the (transformed) NDRs for the KdV soliton condensate.

To find *u*, *v* for the soliton condensate, it is sufficient to invert the FHT *H* on $\Gamma$. Denote by ${\mathcal {R}}_{2N}$ the hyperelliptic Riemann surface of the genus 2*N*, defined by the branchcuts (s-bands) $\gamma _k$, $k=0,\pm 1,\dots , \pm N$, where $\gamma _{-k}=-\gamma _k$. Define two meromorphic differentials of second kind, $\textrm{d}\!p$ and $\textrm{d}q$ on ${\mathcal {R}}_{2N}$ by

3.7 $$\begin{aligned} \textrm{d}p=\frac{iP(\eta )}{2\pi R(\eta )} \textrm{d} \eta , \qquad \textrm{d}q= \frac{2iQ(\eta )}{\pi R(\eta )}\textrm{d}\eta , \end{aligned}$$

where

3.8 $$\begin{aligned} R(\eta )=\sqrt{ (\eta ^2-\lambda _1^2)(\eta ^2-\lambda _2^2)\dots (\eta ^2-\lambda _{2N+1}^2)}, \end{aligned}$$

and *P*, *Q* are odd monic polynomials of degree $2N+1$ and $2N+3$, respectively, that are chosen so that all their s-gap integrals are zero, i.e.

3.9 $$\begin{aligned} \int \limits _{\lambda _{2j-1}}^{\lambda _{2j}} \textrm{d}p = \int \limits _{\lambda _{2j-1}}^{\lambda _{2j}} \textrm{d}q =0, \quad j=1, \dots , N. \end{aligned}$$

Equivalently, one can say that $\textrm{d}p, \textrm{d}q$ are real normalized differentials. Note that Equations (3.7), (3.9) uniquely define $\textrm{d}p,\textrm{d}q$.

### Theorem 3.1

Functions $u(\eta )=\textrm{d}p/\textrm{d}\eta$ and $v(\eta )=\textrm{d}q/\textrm{d}\eta$ defined by (3.7) and (3.9) satisfy the respective equations (3.5) and are odd and real valued on $\Gamma$. Thus, *u*, *v* are the solutions of NDRs (2.9), (2.10) for $\sigma =0$. Moreover, $u(\eta )\ge 0$ on $\Gamma ^+$. Here the value of $R(\eta )$ for $\eta \in \Gamma$ is taken on the positive (upper) shore of the branchcut.

Theorem 3.1 for *u* was proven in Kuijlaars and Tovbis (2021), Sect. 6, for the so-called bound state soliton condensate for the focusing NLS equation. The proof for KdV is analogous. The proof for *v* goes along the same lines, except $v(\eta )$ attains different signs.

Thus, for the soliton condensate of genus *N*, we obtain, on using Theorem 3.1 and Equation (3.7),

3.10 $$\begin{aligned} \begin{aligned}&u(\eta ) \equiv u^{(N)}(\eta ; \lambda _1, \dots \lambda _{2N+1}) = \frac{iP(\eta )}{2\pi R(\eta )}, \\&v(\eta ) \equiv v^{(N)}(\eta ; \lambda _1, \dots , \lambda _{2N+1})= \frac{2iQ(\eta )}{\pi R(\eta )}.\\ \end{aligned} \end{aligned}$$

The velocity of a tracer soliton with the spectral parameter $\eta$ propagating in the soliton condensate with DOS $u(\eta )$ is then found as

3.11 $$\begin{aligned} s(\eta ) \equiv s^{(N)}(\eta ; \lambda _1, \dots , \lambda _{2N+1}) = \frac{v(\eta )}{u(\eta )} = \frac{4Q(\eta )}{P(\eta )}. \end{aligned}$$

**Fig. 1:**
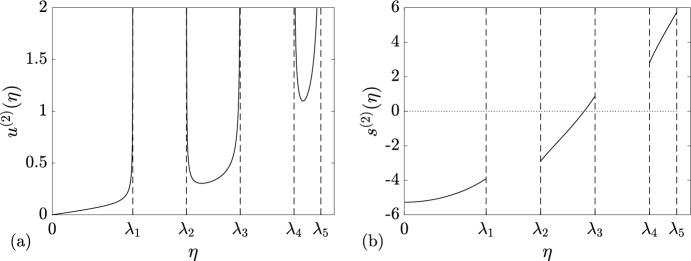
Spectral distributions (3.10) for genus 2 soliton condensate. **a** DOS $u^{(2)}(\eta ; {\varvec{\lambda }})$. **b** Tracer velocity $s^{(2)}(\eta ; {\varvec{\lambda }})$. Here ${\varvec{\lambda }}=(\lambda _1, \lambda _2, \lambda _3, \lambda _4, \lambda _5)=(0.3, 0.5, 0.7, 0.9, 1)$

As an illustrative example we present in Fig. 1, the plots of the DOS and tracer velocity for the genus 2 soliton condensate.

We now consider slow modulations of non-equilibrium (non-uniform) soliton condensates by assuming $u \equiv u( \eta ; x, t)$, $v \equiv v( \eta ; x, t)$, $\Gamma \equiv \Gamma (x,t)$. Equations (2.12), (3.10) then yield the kinetic equation for genus *N* soliton condensate:

3.12 $$\begin{aligned} \left(\frac{P}{R}\right)_t + \left(\frac{4Q}{R}\right)_x =0, \end{aligned}$$

that is valid for $\eta \in \Gamma =\cup _{k=-N}^N (\gamma _k)$. The velocity (3.11) then assumes the meaning of the tracer, or transport, velocity in a non-uniform genus *N* soliton condensate.

### Theorem 3.2

The kinetic equation (3.12) for soliton condensate implies the evolution of the endpoints $\lambda _j$, $j=1,\dots ,2N+1$ according to the Whitham modulation equations

3.13 $$\begin{aligned} \partial _t \lambda _j + V_j({\varvec{\lambda }}) \partial _x \lambda _j = 0, \quad j=1, \dots , 2N+1, \end{aligned}$$

where $${\varvec{\lambda }} = (\lambda _1, \dots , \lambda _{2N+1})$$ and

3.14 $$\begin{aligned} V_j(\varvec{\lambda })=s^{(N)}(\lambda _j; \lambda _1, \dots , \lambda _{2N+1}) = \frac{4Q(\lambda _j)}{P(\lambda _j)}. \end{aligned}$$

### Proof

(See Dubrovin and Novikov 1989) Multiplying (3.12) by $(\eta ^2 - \lambda _j^2)^{3/2}$ and passing to the limit $\eta \rightarrow \lambda _j$, we obtain equations (3.13), (3.14) for the evolution of the spectral s-bands (i.e. the evolution of $\Gamma (x,t)$). These are the KdV–Whitham modulation equations Flaschka et al. (1980), Lax and Levermore (1983) (see also Remark 3.1). $\square$

### Corollary 3.1

The endpoints of the “special” band $\gamma _k=[\lambda _{2k}, \lambda _{2k+1}]$, $k\ne 0$, containing the point $\eta _0$ of zero tracer speed, $s(\eta _0)=0$, are moving in opposite directions, whereas all the endpoints on the same side from $\eta _0$ are moving in the same direction. See Fig. 1 (right) for $N=2$

### Remark 3.1

Modulation equations (3.12), (3.13) were originally derived by Flaschka et al. (1980) by averaging the KdV equation over the multiphase (finite-gap) family of solutions. These equations, along with the condensate NDRs (3.5), also appear in the seminal work of Lax and Levermore (1983) in the context of the semiclassical (zero-dispersion) limit of multi-soliton KdV ensembles (see Sect. 5 and, in particular, Equation (5.23) in Lax and Levermore (1983)). A succinct exposition of the spectral Whitham theory for the KdV equation can be found in Dubrovin and Novikov (1989)).

### Remark 3.2

The Whitham modulation equations (3.13), (3.14) are locally integrable for any *N* via Tsarev’s generalized hodograph transform (Tsarëv 1991; Dubrovin and Novikov 1989). Moreover, by allowing the genus *N* to take different values in different regions of *x*, *t*-plane, $N=N(x,t)$, global solutions of the KdV–Whitham system can be constructed for a broad class of initial data (see Sect. 4.2 for further details). Invoking the definitive property $\sigma \equiv 0$ of a soliton condensate, the existence of the solution to an initial value problem for the Whitham system for all $t>0$ implies that this property will remain invariant under the *t*-evolution, i.e. soliton condensate will remain a condensate during the evolution; however, its genus can change.

The finite-genus Whitham modulation system (3.13), (3.14) can be viewed as an exact hydrodynamic reduction of the full kinetic equation (2.11), (2.12) under the ansatz (3.10), (3.11). Recalling the origin of the soliton gas kinetic equation as a singular, thermodynamic limit of the Whitham equations (El 2003), the recovery of the finite-genus Whitham dynamics in the condensate limit might not look surprising. On the other hand, viewed from the general soliton gas perspective the condensate reduction notably shows that the highly nontrivial nonlinear modulation (hydro)dynamics emerges as a collective effect of the elementary two-soliton scattering events. This understanding is in line with ideas of generalized hydrodynamics, a powerful theoretical framework for the description of non-equilibrium macroscopic dynamics in many-body quantum and classical integrable systems. The connection of the KdV soliton gas theory with generalized hydrodynamics has been recently established in Bonnemain et al. (2022). Relevant to the above, it was shown in that the semiclassical limit of the generalized hydrodynamics for the Lieb–Liniger model of Bose gases yields the Whitham modulation system for the defocusing NLS equation.

A different type of hydrodynamic reductions of the soliton gas kinetic equation realized via the multi-component delta-function ansatz $u(\eta ; x,t)= \sum _{i=1}^{m} w_i(x,t)\delta (\eta -\eta _j)$ for the DOS has been studied in El et al. (2011) for $\eta _j = \textrm{const}$ and in Pavlov et al. (2012); Ferapontov and Pavlov (2022) for $\eta _j =\eta _j(x,t)$. One of the defining properties of the multicomponent hydrodynamic reductions of this type is their linear degeneracy which, in particular, implies the absence of wavebreaking and the occurrence of contact discontinuities in the solutions of Riemann problems  (Smoller 1994) studied in the context of two-component KdV soliton gases in Carbone et al. (2016). In contrast, the condensate (Whitham) system (3.13), (3.14) obtained under the condition $\sigma \equiv 0$ is known to be *genuinely nonlinear*, $\partial V_j /\partial \lambda _j \ne 0$, $j=1, \dots 2N+1$ (Levermore 1988) implying the inevitability of wavebreaking for general initial data, which is in stark contrast with linear degeneracy of the multicomponent “cold-gas” hydrodynamic reductions. Reconciling the genuine nonlinearity property of the Whitham equations governing soliton condensates with linearly degenerate non-condensate multicomponent cold-gas dynamics is an interesting problem, which will be considered in future publications.

Thus, we have shown that the spectral dynamics of non-equilibrium soliton condensates are equivalent to those of modulated finite gap potentials, which naturally suggests a close connection between (or even equivalence of) these two objects at the level of realizations, i.e. the corresponding solutions $\varphi (x,t)$ of the KdV equation itself. This connection will be explored in the next section using a combination of analytical results and numerical simulations for genus 0 and genus 1 soliton condensates.

## Genus 0 and Genus 1 Soliton Condensates

Having developed the spectral description of KdV soliton condensates, we now look closer at the two simplest representatives: genus 0 and genus 1 condensates. In particular, we shall be interested in the characterization of the *realizations* of soliton condensates, i.e. the KdV solutions, denoted $\varphi _\textrm{c}^{(N)}(x,t)$, corresponding to the condensate spectral DOS $u^{(N)}(\eta )$ for $N=0,1$. We do not attempt here to construct the soliton gas realizations explicitly via the thermodynamic limit of finite gap potentials (see Sect. 2); instead, we infer some of their key properties from the expressions (3.4) for the ensemble averages as moments of the spectral DOS. We then conjecture the exact form of soliton condensate realizations and support our conjecture by comparison with detailed numerical simulations.

### Equilibrium Properties

#### Genus 0

For $N=0$, equations (3.10) for the DOS and the spectral flux density yield (cf. (2.16))

4.1 $$\begin{aligned} u(\eta )=u^{(0)}(\eta ; \lambda _1) \equiv \frac{\eta }{\pi \sqrt{\lambda _1^2-\eta ^2}},\quad v(\eta )= v^{(0)}(\eta ;\lambda _1) \equiv \frac{6\eta (2\eta ^2-\lambda _1^2)}{\pi \sqrt{\lambda _1^2-\eta ^2}}, \end{aligned}$$

so that the tracer velocity (cf. (3.11))

4.2 $$\begin{aligned} s(\eta )=s^{(0)}(\eta ;\lambda _1) = 6(2\eta ^2-\lambda _1^2). \end{aligned}$$

**Fig. 2:**
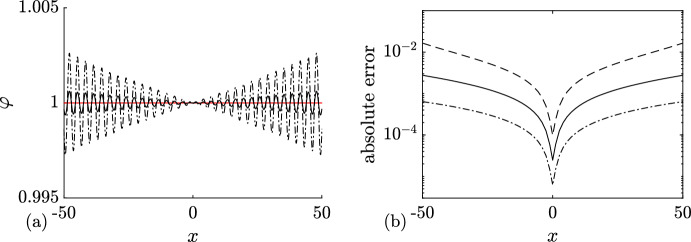
**a** Comparison between numerical realizations of genus 0 condensate generated with 100 solitons (dashed line), 200 solitons (black solid line), and the constant KdV solution $\varphi =1$ (red solid line). **b** Corresponding absolute errors $|\varphi _n(x)-1|$ obtained with 50 solitons (dashed line), 100 solitons (solid line) and 200 (dash-dotted line); the absolute error is evaluated at the extrema of the oscillations (Color figure online)

Next, substituting (4.1) in (3.4) (where $\Gamma = [-\lambda _1,\lambda _1]$ or, equivalently, $\Gamma ^+ = [0,\lambda _1]$), we obtain for the ensemble averages:

4.3 $$\begin{aligned} \langle \varphi \rangle = \lambda _1^2,\quad \langle \varphi ^2 \rangle = \lambda _1^4, \end{aligned}$$

where $\varphi \equiv \varphi _\textrm{c}^{(0)}(x,t)$. Thus, the variance $\Delta =\sqrt{\langle \varphi ^2 \rangle - \langle \varphi \rangle ^2}=\sqrt{\langle ( \varphi - \langle \varphi \rangle )^2 \rangle }= 0$, which implies (see, e.g. Rohatgi and Ehsanes Saleh 2015) that genus 0 soliton condensate is *almost surely* described by a constant solution of the KdV equation, i.e.

4.4 $$\begin{aligned} \varphi = \langle \varphi \rangle = \lambda _1^2 \end{aligned}$$

(note that constant solution is classified as a genus 0 KdV potential).

**Fig. 3:**
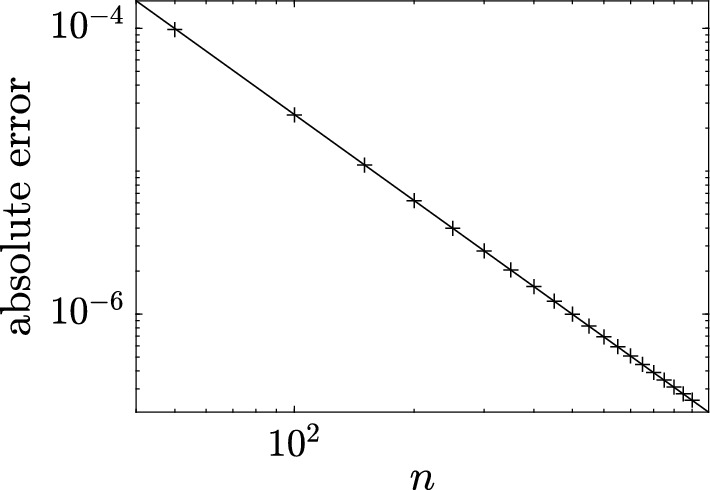
Variation of the absolute error $|\varphi _n(x)-1|$ at the center of the numerical domain $x=0$ (cf. Figure 2). The markers correspond to the error obtained numerically, and the solid line the corresponding fit $\alpha /n^2$ where $\alpha \approx 0.25$

This result can be intuitively understood by identifying soliton condensate with the “densest possible” soliton gas for a given spectral support $\Gamma$. The densest “packing” for genus 0 is achieved by distributing soliton parameters according to the spectral DOS $u(\eta )$ (4.1), which results in the individual solitons “merging” into a uniform KdV field of amplitude $\lambda _1^2$. The numerical implementation of soliton condensate realizations, using *n*-soliton KdV solution with *n* large, shows that the condensate DOS (4.1) is only achievable within this framework if all *n* solitons in the solution have the same phase of the respective norming constants. Invoking the interpretation of the phase of the norming constant as the soliton position in space (Novikov et al. 1984; Drazin and Johnson 1989), one can say that in the condensate all solitons are placed at the same point, say $x=0$ (cf. Appendix A for a mathematical justification). Details of the numerical implementation of KdV soliton gas using *n*-soliton solutions can be found in Appendix A. Figure 2 displays the realization $\varphi _\textrm{c}^{(0)}(x)$ of genus 0 soliton condensate with $\lambda _1=1$ modeled by *n*-soliton solutions $\varphi _n(x)$ with $n=100$ and $n=200$, along with the absolute errors $\varphi _n(x)-1$; in the following we refer to these *n*-soliton solutions as “numerical realizations” of the soliton gas. One can see that the error at the center of the numerical domain, where the gas is nearly uniform, is very small: Fig. 3 displays the variation of this error with *n* and shows that it decreases with $1/n^2$.

The numerical approximation used here is similar to the approximation of the soliton condensate of the focusing NLS equation via a *n*-soliton solution presented in Gelash et al. (2021). In the latter case, the uniform wavefield limit, which can be viewed as a central part of the so-called box potential, is also reached when the complex phases of the norming constants are chosen deterministically. The absolute error—the difference between the focusing NLS *n*-soliton solution and the expected constant value of the wavefield—measured at the center of the numerical realization—follows a different scaling law and is proportional to $n^{-1/2}$.

#### Genus 1

We now consider the case of genus 1 soliton condensate. For $N=1$

4.5 $$\begin{aligned} R(\eta )=\sqrt{(\eta ^2-\lambda _1^2)(\eta ^2-\lambda _2^2)(\eta ^2-\lambda _3^2)} \end{aligned}$$

is purely imaginary on $\Gamma =[-\lambda _3,-\lambda _2]\cup [-\lambda _1,\lambda _1]\cup [\lambda _2,\lambda _3]$. According toTheorem 3.1

4.6 $$\begin{aligned}&u(\eta )=u^{(1)}(\eta ;\lambda _1,\lambda _2,\lambda _3) \equiv \frac{i\eta (\eta ^2-w^2)}{\pi R(\eta )}, \end{aligned}$$

4.7 $$\begin{aligned}&v(\eta )=v^{(1)}(\eta ;\lambda _1,\lambda _2,\lambda _3) \equiv \frac{12i\eta (\eta ^4-h^2\eta ^2-r^2)}{\pi R(\eta )}, \end{aligned}$$

where $h^2=\frac{\lambda _1^2+\lambda _2^2+\lambda _3^2}{2}$ follows from the fact that $\left. -i\textrm{Res}\frac{Q(\zeta )}{(\zeta -\eta )R(\zeta )}\right| _{\zeta =\infty }=-6\eta ^2$. The normalization conditions (3.9) imply that

4.8 $$\begin{aligned} w^2=\dfrac{\int _{\lambda _1}^{\lambda _2}\frac{y^3\textrm{d}y}{R(y)}}{\int _{\lambda _1}^{\lambda _2}\frac{y\textrm{d}y}{R(y)}},\quad r^2= \dfrac{\int _{\lambda _1}^{\lambda _2}\frac{y^5-\frac{\lambda _1^2+\lambda _2^2+\lambda _3^2}{2}y^3}{R(y)}\textrm{d}y}{\int _{\lambda _1}^{\lambda _2}\frac{y\textrm{d}y}{R(y)}}. \end{aligned}$$

Using 3.131.3 and 3.132.2 from Gradshteyn and Ryzhik (2007), we calculate

4.9 $$\begin{aligned} w^2\!=\!\lambda _3^2-(\lambda _3^2-\lambda _1^2)\chi (m), \quad \textrm{where}\quad \chi (m)\!=\!\frac{\textrm{E} \left( m \right) }{\textrm{K} \left( m \right) } \quad \textrm{and}\quad m \!=\! \frac{\lambda _2^2\!-\!\lambda _1^2}{\lambda _3^2\!-\!\lambda _1^2}. \end{aligned}$$

Calculation of $r^2$ is a bit more involved as it is based on the observation

4.10 $$\begin{aligned} \begin{aligned} \int _{\lambda _1}^{\lambda _2} \frac{y^5}{R(y)}\textrm{d}y&=\frac{1}{2}\int _{\lambda _1^2}^{\lambda _2^2}\frac{z^2\textrm{d}z}{R(z^\frac{1}{2})}, \\&= \frac{\lambda _1^2+\lambda _2^2+\lambda _3^2}{3}\int _{\lambda _1^2}^{\lambda _2^2}\frac{z\textrm{d}z}{R(z^\frac{1}{2})}-\frac{\lambda _1^2\lambda _2^2+\lambda _1^2\lambda _3^2+\lambda _2^2\lambda _3^2}{6}\int _{\lambda _1^2}^{\lambda _2^2}\frac{\textrm{d}z}{R(z^\frac{1}{2})}. \end{aligned}\nonumber \\ \end{aligned}$$

Using (4.8), (4.10), we obtain after some algebra

4.11 $$\begin{aligned} r^2=\frac{1}{6}\left[ \lambda _3^2(\lambda _3^2-\lambda _2^2-\lambda _1^2)-2\lambda _2^2\lambda _1^2 - (\lambda _3^2+\lambda _1^2+\lambda _2^2)(\lambda _3^2-\lambda _1^2)\chi (m)\right] . \end{aligned}$$

Thus, the velocity of a tracer soliton with spectral parameter $\eta \in \Gamma ^+$ in the genus 1 soliton condensate, characterized by DOS (4.6), is given by

4.12 $$\begin{aligned} \begin{aligned} s(\eta )&\equiv s^{(1)}(\eta ;\lambda _1,\lambda _2,\lambda _3) = 12\frac{\eta ^4- \frac{\lambda _2^2+\lambda _3^2+\lambda _1^2}{2}\eta ^2-r^2}{\eta ^2-w^2}\\&= 12\frac{\eta ^4- \frac{\lambda _2^2+\lambda _3^2+\lambda _1^2}{2}\eta ^2-\frac{\lambda _3^2(\lambda _3^2-\lambda _2^2-\lambda _1^2)-2\lambda _2^2\lambda _1^2 - (\lambda _3^2+\lambda _1^2+\lambda _2^2)(\lambda _3^2-\lambda _1^2)\chi (m)}{6}}{\eta ^2-\lambda _3^2+(\lambda _3^2-\lambda _1^2)\chi (m)}. \end{aligned}\nonumber \\ \end{aligned}$$

We note that a similar expression for the tracer velocity in a dense soliton gas was obtained in Girotti et al. (2022) in the context of the modified KdV (mKdV) equation.

For $N=1$, the integrals (3.4) for the mean and mean square of the soliton condensate wave field $\varphi \equiv \varphi _\textrm{c}^{(1)}(x,t)$ can be explicitly evaluated using (253.11) and (256.11) from Byrd and Friedman (1954) and 19.7.10 from Olver et al. (2022):

4.13 $$\begin{aligned}&\langle \varphi \rangle = \lambda _1^2+\lambda _2^2-\lambda _3^2+2(\lambda _3^2-\lambda _1^2) \chi (m), \end{aligned}$$

4.14 $$\begin{aligned}&\langle \varphi ^2 \rangle = \frac{2(\lambda _1^2+\lambda _2^2+\lambda _3^2)}{3} \langle \varphi \rangle +\frac{\lambda _1^4+\lambda _2^4+\lambda _3^4-2\lambda _1^2\lambda _2^2-2\lambda _2^2\lambda _3^2-2\lambda _1^2\lambda _3^2}{3}, \end{aligned}$$

with $\chi (m)$ and *m* given by (4.9). It is not difficult to verify that, unlike in the case of genus 0 condensates, the variance $\Delta =\sqrt{\langle \varphi ^2 \rangle - \langle \varphi \rangle ^2}$ does not vanish identically, implying that all realizations of the genus 1 soliton condensate are almost surely non-constant.

A key observation is that formulae (4.13), (4.14) coincide with the period averages ${\overline{\varphi }}$ and $\overline{\varphi ^2}$ of the genus 1 KdV solution associated with the spectral Riemann surface ${\mathcal {R}}_2$ of (4.5) (see, e.g. Kamchatnov 2000; El and Hoefer 2016):

4.15 $$\begin{aligned}{} & {} \varphi (x,t) \equiv F_1(\theta ; \lambda _1, \lambda _2, \lambda _3)= \lambda _1^2+\lambda _2^2-\lambda _3^2 +2(\lambda _3^2-\lambda _1^2)\textrm{dn}^2 \left( \frac{\sqrt{\lambda _3^2-\lambda _1^2}}{k} \theta ;m \right) ,\nonumber \\{} & {} \theta = k(x-Ut) +\theta ^0,\quad U=2(\lambda _1^2+\lambda _2^2+\lambda _3^2), \quad k= \frac{\pi \sqrt{\lambda _3^2 - \lambda _1^2}}{\textrm{K}(m)}, \end{aligned}$$

where $\theta ^0 \in [0, 2\pi )$ is an arbitrary initial phase.

The equivalence between the ensemble averages in genus 1 KdV soliton condensates and the period averages in single-phase KdV solutions, along with the established in Sect. 3 equivalence between the respective modulation dynamics, strongly suggest that realizations of the genus 1 soliton condensates are described by the periodic solutions $F_1(\theta )$ (4.15) of the KdV equation. This motivates the following

##### Conjecture 4.1

For any realization $\varphi =\varphi _\textrm{c}^{(1)}(x,t)$ of the genus 1 KdV soliton condensate associated with the spectral curve ${\mathcal {R}}_2$ of (4.5), one can find the initial phase $\theta ^0 \in [0, 2 \pi )$ in the periodic solution $F_1 (\theta ; \lambda _1, \lambda _2, \lambda _3)$ (4.15) such that almost surely $\varphi _\textrm{c}^{(1)}(x,t) = F_1 (\theta ; \lambda _1, \lambda _2, \lambda _3)$.

**Fig. 4:**
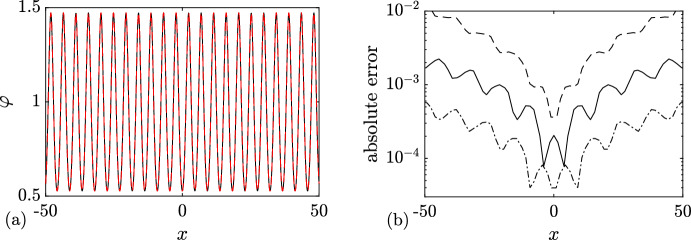
**a** Comparison between the numerical realization of genus 1 condensate generated with 200 solitons (black solid line), 200 solitons (black solid line), and the exact cnoidal wave solution $F_1(kx)$ (4.15) (red dashed line) for $\lambda _1=0.5,\lambda _2=0.85,\lambda _3=1$ ($m=0.63$); the two plots are visually indistinguishable from one another; **b** the corresponding absolute errors $\varphi _n(x)-F_1(kx)$ obtained with 50 solitons (dashed line), 100 solitons (solid line) and 200 (dash-dotted line); the absolute error is evaluated at the extrema of the oscillations (Color figure online)

We support Conjecture 4.1 by a detailed comparison of a numerical realization of KdV soliton condensate (as *n*-soliton solution with *n* large) spectrally configured according to the DOS (4.6), and the periodic KdV solution (4.15), defined on the same spectral curve ${\mathcal {R}}_2$, with the appropriately chosen initial phase $\theta ^0$ (see Appendix A for the details of the numerical implementation of soliton condensate). The comparison is presented in Fig. 4 and reveals a remarkable agreement, which further improves as *n* increases.

Conjecture 4.1 can be naturally generalized to an arbitrary genus *N*: for any realization of the KdV soliton condensate of genus *N* corresponding to the DOS $u^{(N)}(\eta ; {\varvec{\lambda }})$ (3.10) and associated with the spectral Riemann surface ${\mathcal {R}}_{2N}$ of (3.8), one can find *N*-component initial phase vector ${\varvec{\theta }}^0 \in {\mathbb {T}}^N$ so that $\varphi ^{(N)}_\textrm{c}(x,t)$ almost surely coincides with *N*-phase KdV solution $\varphi =F_N({\varvec{\theta }}; {\varvec{\lambda }})$ (2.5). To support this generalization, we performed a comparison of a numerical realization of the genus 2 soliton condensate with the respective two-phase (two-gap) KdV solution, see Appendix B.

A rigorous mathematical proof of Conjecture 4.1 and its generalization for an arbitrary genus will be the subject of future work.

In conclusion, we note that Conjecture 4.1 correlates with the results of Girotti et al. (2021) where a particular “deterministic soliton gas” solution of the KdV equation was constructed by considering the *n*-soliton solution with the discrete spectrum confined within two symmetric intervals—the analogs of s-bands of our work—and letting $n \rightarrow \infty$. This solution was shown in Girotti et al. (2021) to represent a *primitive potential* (Dyachenko et al. 2016) whose long-time asymptotics is described at leading order by a modulated genus 1 KdV solution. A similar construction was realized in Girotti et al. (2022) for the mKdV equation. We also mention the recent paper (Nabelek 2020) where the direct connection between finite-gap KdV solutions and primitive potentials was established.

### Modulation Dynamics

The dynamics of DOS in non-equilibrium (weakly non-homogeneous) soliton condensates is determined by the evolution of the endpoints $\lambda _j$ of the spectral bands of $\Gamma$ (the s-bands). As proven in Sect. 3, this evolution is governed by the Whitham modulation equations (3.13). Properties of the KdV–Whitham modulation systems are well studied: in particular, system (3.13) is strictly hyperbolic and genuinely nonlinear for any genus $N \ge 1$ (Levermore 1988). This implies inevitability of wavebreaking for a broad class of initial conditions. What is the meaning of the wavebreaking in the context of soliton condensates, and how is the solution of the kinetic equation for the condensate continued beyond the wavebreaking point in the (*x*, *t*)-plane?

We first invoke the definitive property of a soliton condensate—the vanishing of the spectral scaling function, $\sigma (\eta ) \equiv 0$, in the soliton gas NDRs (2.9). According to Remark 3.2, if $\sigma (\eta ; x, 0) \equiv 0$ for all $x \in {\mathbb {R}}$, then $\sigma (\eta ; x, t) \equiv 0$ for all $x \in {\mathbb {R}}$, $\forall t>0$, implying that soliton condensate necessarily remains a condensate during the evolution (at least of some class of initial data). The only qualitative modification that is permissible during the evolution is the change of the genus *N*. The description of the evolution of a soliton condensate is then reduced to the determination of the spectral support $\Gamma (x,t)$, parametrizing the DOS via the band edges $\lambda _j(x,t)$: $u=u^{(N)}(\eta ; \lambda _1, \dots \lambda _{2N+1})$ (3.10).

In view of the above, the evolution of soliton condensates can be naturally put in the framework of the problem of hydrodynamic evolution of multivalued functions originally formulated by Dubrovin and Novikov (1989). Let $\Lambda _N (x,t)=\{\lambda _1(x,t), \dots , \lambda _{2N+1} (x,t)\}$ be a smooth multivalued curve whose branches $\lambda _j (x,t)$ satisfy the Whitham modulation equations (3.13). Then, if the wavebreaking occurs within one of the branches, it results in a change of the genus *N* so that $\Lambda _{N} \rightarrow \Lambda _{N+1}$ in some space-time region $[x^-(t), x^+(t)]$ that includes the wavebreaking point. The curves $\Lambda _N$ and $\Lambda _{N+1}$ are glued together at free boundaries $x^{\pm }(t)$. Details of the implementation of this procedure can be found in (Dubrovin and Novikov 1989; Dubrovin 1997; El et al. 2001; Grava and Tian 2002). The simplest case of the multivalued curve evolution arises when the initial data for $\Lambda _N$ is a piecewise-constant distribution (both for $\lambda _j$’s and for *N*), with a discontinuity at $x=0$—a Riemann problem. In this special case, the wavebreaking occurs at $t=0$ (subject to appropriate sign of the initial jump) and smoothness of $\Lambda _N$ is not a prerequisite.

In this paper, we restrict ourselves to Riemann problems involving only genus 0 and genus 1 modulation solutions and show how the resulting spectral dynamics are interpreted in terms of soliton condensates. For that, we will need explicit expressions for the Whitham characteristic velocities for $N=0$ and $N=1$. These expressions are known very well (see, e.g. Gurevich and Pitaevskii 1974; Kamchatnov 2000; El and Hoefer 2016), but here we obtain them as transport velocities for the respective soliton condensates, using the expressions (4.2), and (4.12), respectively.

(i) $N=0$. Consider a non-equilibrium (non-uniform) soliton condensate of genus 0, characterized by a space-time-dependent DOS $u(\eta ; x, t)$. To this end, we set $\eta = \lambda _1(x,t)$ in (4.2); then, the Whitham system (3.13), (3.14) assumes the form of the Hopf (inviscid Burgers) equation

4.16 $$\begin{aligned} (\lambda _1)_t +6\lambda _1^2(\lambda _1)_x =0. \end{aligned}$$

Note that this is exactly the result obtained by Lax and Levermore (1983) for the pre-breaking evolution of semi-classical soliton ensembles.

(ii) $N=1$. We obtain on using (4.12),

4.17 $$\begin{aligned} (\lambda _j)_t + V_j(\lambda _1,\lambda _2,\lambda _3) (\lambda _j)_x = 0,\quad j=1,2,3, \end{aligned}$$

where

4.18 $$\begin{aligned} \begin{aligned} V_1(\lambda _1,\lambda _2,\lambda _3) \equiv&\, s^{(1)}(\lambda _1;\lambda _1,\lambda _2,\lambda _3) = 2(\lambda _1^2+\lambda _2^2+\lambda _3^2)+\frac{4(\lambda _2^2-\lambda _1^2)}{\chi (m)-1}, \\ V_2(\lambda _1,\lambda _2,\lambda _3) \equiv&\, s^{(1)}(\lambda _2;\lambda _1,\lambda _2,\lambda _3) = 2(\lambda _1^2+\lambda _2^2+\lambda _3^2) \\ +&\,\frac{4(\lambda _3^2-\lambda _2^2)(\lambda _2^2-\lambda _1^2)}{\lambda _3^2-\lambda _2^2-(\lambda _3^2-\lambda _1^2)\chi (m)}, \\ V_3(\lambda _1,\lambda _2,\lambda _3) \equiv&\, s^{(1)}(\lambda _3;\lambda _1,\lambda _2,\lambda _3) = 2(\lambda _1^2+\lambda _2^2+\lambda _3^2) + \frac{4(\lambda _3^2-\lambda _2^2)}{\chi (m)}, \end{aligned} \end{aligned}$$

and $\chi (m)$ is defined in (4.9). System (4.17), (4.18) coincides with the original Whitham modulation equations derived for $r_j =6\lambda _j^2$ in Whitham (1965) by averaging KdV conservation laws over the single-phase, cnoidal wave family of solutions (see also Gurevich and Pitaevskii 1974; Dubrovin and Novikov 1989; Kamchatnov 2000; El and Hoefer 2016).

## Riemann Problem for Soliton Condensates

The classical Riemann problem consists of finding solution to a system of hyperbolic conservation laws subject to piecewise-constant initial conditions exhibiting discontinuity at $x = 0$. The distribution solution of such Riemann problem generally represents a combination of constant states, simple (rarefaction) waves and strong discontinuities (shocks or contact discontinuities) (Lax 1973). In dispersive hydrodynamics, classical shock waves are replaced by dispersive shock waves (DSWs)—nonlinear expanding wavetrains with a certain, well-defined structure (El and Hoefer 2016). Here we generalize the Riemann problem formulation to the soliton gas kinetic equation by considering (1.1) subject to discontinuous initial DOS:

5.1 $$\begin{aligned} u(\eta ; x,t=0) = {\left\{ \begin{array}{ll} u^{(N_-)}(\eta ;\lambda _1^-, \dots , \lambda _{2N_-+1}^-), &{} x<0, \\ u^{(N_+)}(\eta ;\lambda _1^+, \dots , \lambda _{2N_++1}^+), &{} x>0, \end{array}\right. } \end{aligned}$$

where $u^{(N)}(\eta ; \lambda _1, \dots , \lambda _{2N+1})$ is the DOS (3.10) of genus *N* condensate and $\lambda _j^\pm >0$.

As discussed in Sect. 4.2, soliton condensate necessarily retains its definitive property $\sigma =0$ during the evolution, with the only qualitative modification permissible being the change of the genus *N*. The evolution of the soliton condensate is then determined by the motion of the s-band edges $\lambda _j$ according to the Whitham modulation equations (3.13) subject to discontinuous initial conditions following from (5.1):

5.2 $$\begin{aligned} \{N; {\varvec{\lambda }}\}( x,t=0) = {\left\{ \begin{array}{ll} \{N_-; (\lambda _1^-, \dots , \lambda _{2N_-+1}^-) \}, &{} x<0, \\ \{N_+; (\lambda _1^+, \dots , \lambda _{2N_+ +1}^+)\}, &{} x>0. \end{array}\right. } \end{aligned}$$

Thus, the Riemann problem for soliton gas kinetic equation is effectively reduced in the condensate limit to the Riemann problem (5.2) for the Whitham modulation equations (3.13). Depending on the sign of the jump $\lambda _j^- - \lambda _j^+$, the regularization of the discontinuity in $\lambda _j$ can occur in two ways: (i) if $(\lambda _j^- - \lambda _j^+)<0$, then the regularization occurs via the generation of a rarefaction wave for $\lambda _j$ without changing the genus *N* of the condensate; (ii) if $(\lambda _j^- - \lambda _j^+)>0$ (which implies immediate wavebreaking for $\lambda _j$), the regularization occurs via the generation of a higher genus condensate whose evolution is governed by the modulation equations.

Below we consider several particular cases of Riemann problems describing some prototypical features of the soliton condensate dynamics.

### $N_-=N_+=0$

Consider the initial condition for the kinetic equation in the form of a discontinuous genus 0 condensate DOS,

5.3 $$\begin{aligned} u(\eta ; x,t=0) = {\left\{ \begin{array}{ll} u^{(0)}(\eta ;q_-), &{} x<0, \\ u^{(0)}(\eta ;q_+), &{} x>0, \end{array}\right. } \end{aligned}$$

where $q_\pm = \lambda _1^\pm$, and $u^{(0)}>0$ is defined in (4.1). The DOS distribution (5.3) implies the step initial conditions for the Whitham modulation system (3.13):

5.4 $$\begin{aligned} N(x,t=0)=0, \quad \lambda _1(x,t=0) = {\left\{ \begin{array}{ll} q_-, &{} x<0, \\ q_+, &{} x>0, \end{array}\right. } \end{aligned}$$

with $q_- \ne q_+$. Additionally, since the wave field in a genus 0 soliton condensate is almost surely a constant, $\varphi (x,t)=(\lambda _1)^2$, we conclude that the DOS distribution (5.3) gives rise to the Riemann step data

5.5 $$\begin{aligned} \varphi (x,t=0) = {\left\{ \begin{array}{ll} q_-^2, &{} x<0, \\ q_+^2, &{} x>0, \end{array}\right. } \end{aligned}$$

for the KdV equation (2.1) itself.

The Riemann problem for the KdV equation was originally studied by Gurevich and Pitaevskii (GP) (Gurevich and Pitaevskii 1974) in the context of the description of dispersive shock waves. The key idea of GP construction was to replace the dispersive Riemann problem (5.5) for the KdV equation by an appropriate boundary value problem for the hyperbolic KdV–Whitham system (4.17), which is then solved in the class of *x*/*t*-self-similar solutions. Here we take advantage of the GP modulation solutions and their higher genus analogues to describe dynamics of soliton condensates. The choice of the genus of the Whitham system and, correspondingly, the genus of the associated soliton condensate, depends on whether $q_-<q_+$ or $q_- > q_+$.

#### Rarefaction Wave ($q_-<q_+$)

The solution of the Riemann problem (1.1), (5.3) is given globally (for $t>0$) by the genus 0 DOS $u^{(0)}(\eta ; \lambda _1)$ (4.1) modulated by the centered rarefaction wave solution of the Hopf equation (4.16) subject to the step initial condition (5.4):

5.6 $$\begin{aligned} \lambda _1(x,t) = {\left\{ \begin{array}{ll} q_-, &{} x<s_-t, \\ \sqrt{\frac{x}{6t}}, &{} s_-t< x< s_+ t, \\ q_+, &{} s_+t<x, \end{array}\right. } \end{aligned}$$

where

5.7 $$\begin{aligned} s_- = 6q_-^2,\quad s_+ = 6q_+^2. \end{aligned}$$

**Fig. 5:**
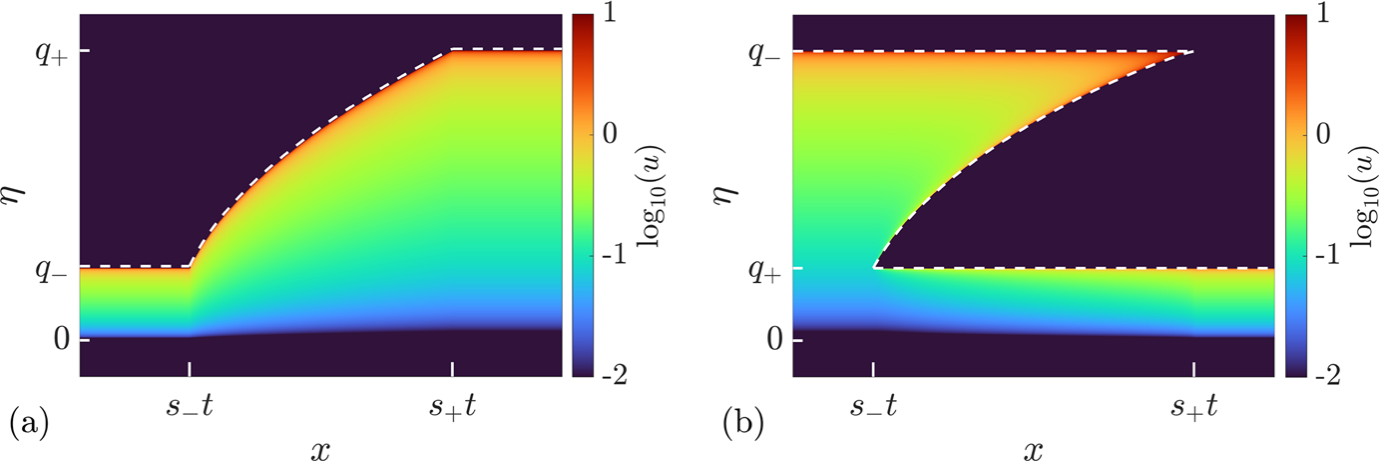
Solutions to the soliton condensate Riemann problem (5.3). **a** Rarefaction wave (genus 0) solution (5.6), (5.8) for $q_- < q_+$. Dashed line: $\lambda _1(x,t)$, colors: DOS $u^{(0)}(\eta ; \lambda _1(x,t))$. **b** DSW (genus 1) solution (5.9), (5.10) for $q_->q_+$. Dashed line: $\lambda _1(x,t) \le \lambda _2(x,t) \le \lambda _3(x,t)$, colors: DOS $u^{(1)}(\eta ; q_+, \lambda _2(x,t), q_-) (Color figure online)$

Note that the solution (5.6) is admissible since $s_-<s_+$. Behavior of $\lambda _1$ in the solution (5.6) is shown in Fig. 5a. The evolution of the soliton condensate’s DOS associated with the spectral rarefaction wave solution (5.6) is given by:

5.8 $$\begin{aligned} u(\eta ; x,t) = \frac{\eta }{\pi \sqrt{\lambda _1^2 (x,t)-\eta ^2}}. \end{aligned}$$

A contour plot of the DOS (5.8) is presented in Fig. 5(a).

#### Dispersive Shock Wave ($q_->q_+$)

The solution (5.6), (5.8) derived previously is not admissible for $q_->q_+$ since$s_->s_+$ in that case. In other words, the compressive discontinuous initial data (5.4) imply immediate wavebreaking and necessitate the introduction of the higher genus DOS connecting $u^{(0)}(\eta ;q_-)$ and $u^{(0)}(\eta ; q_+)$. The requisite DOS is given by equation (4.6), which we reproduce here for convenience,

5.9 $$\begin{aligned} u(\eta ; x,t) = u^{(1)}(\eta ;\lambda _1,\lambda _2,\lambda _3) = \frac{i\eta (\eta ^2-w^2)}{\pi R(\eta )}, \end{aligned}$$

Here $w(\lambda _1, \lambda _2, \lambda _3)$ is given by (4.9) and $\lambda _j=\lambda _j(x,t)$, $j=1,2,3$, are slowly modulated according to the Whitham equations (4.17), (4.18).

The solution of (4.18) is self-similar, $\lambda _j(x/t)$, such that $u^{(1)}(\eta ;\lambda _1,\lambda _2,\lambda _3)$ matches with $u^{(0)}(\eta ;q_-)$ at the left boundary $x=s_-t$, and with $u^{(0)}(\eta ;q_+)$ at the right boundary $x=s_+t$, with $s_-<s_+$.

The requisite solution is the 2-wave of the Whitham system (4.17) (only $\lambda _2$ is non-constant)

5.10 $$\begin{aligned} \lambda _1 = q_+,\quad V_2(\lambda _1,\lambda _2,\lambda _3) = x/t,\quad \lambda _3=q_-,\quad \text {for}\quad s_-t<x<s_+ t, \end{aligned}$$

where

5.11 $$\begin{aligned}{} & {} s_- = V_2(q_+,q_+,q_-) = 12q_+^2-6q_-^2,\nonumber \\{} & {} s_+ = V_2(q_+,q_-,q_-) = 2q_+^2+4q_-^2. \end{aligned}$$

This is the famous GP solution describing the DSW modulations in the KdV step resolution problem (Gurevich and Pitaevskii 1974). Indeed, we have $s_-<s_+$, and interpreting the GP solution (5.10) in terms of soliton condensates, the limiting behaviors at the DSW edges are given by:

5.12 $$\begin{aligned} \begin{aligned} \begin{aligned}x \rightarrow s_-t,\quad \lambda _2 \rightarrow \lambda _1=q_+,\quad u^{(1)}(\eta ;q_+,\lambda _2,q_-) \rightarrow u^{(0)}(\eta ;q_-),\\x \rightarrow s_+t,\quad \lambda _2 \rightarrow \lambda _3=q_-,\quad u^{(1)}(\eta ;q_+,\lambda _2,q_-) \rightarrow u^{(0)}(\eta ;q_-). \end{aligned} \end{aligned} \end{aligned}$$

### $N_-+N_+=1$

Before considering the soliton condensate Riemann problem (1.1), (5.1) for the case $N_-+N_+=1$, we list the admissible solutions to the kinetic equation connecting a genus 0 distribution $u^{(0)}(\eta ;q)$ to a genus 1 distribution $u^{(1)}(\eta ;\lambda _1,\lambda _2,\lambda _3)$. One can easily verify for the next four solutions that

5.13 $$\begin{aligned} \begin{aligned}&x \rightarrow s_-t,\quad u^{(1)}(\eta ;\lambda _1,\lambda _2,\lambda _3) \rightarrow u^{(N_-)}(\eta ; \varvec{\lambda }_-),\\&x \rightarrow s_+t,\quad u^{(1)}(\eta ;\lambda _1,\lambda _2,\lambda _3) \rightarrow u^{(N_+)}(\eta ; \varvec{\lambda }_+), \end{aligned} \end{aligned}$$

with $$s_-<s_+$$.

We use the following convention to label the fundamental Riemann problem solutions: we call $j^\pm$-wave, where *j* is the index of the only varying Riemann invariant $\lambda _j$ in the solution, while the remaining invariants are constant; $+$ indicates that $N_+=1$, i.e., the genus 1 soliton condensate is initially at $x>0$, and − indicates that $N_-=1$, i.e., the genus 1 soliton condensate is initially at $x<0$.

(i) $$3^+$$-*wave*

Consider the initial condition for the soliton condensate DOS:

5.14 $$\begin{aligned} u(\eta ; x,t=0) = {\left\{ \begin{array}{ll} u^{(0)}(\eta ;q_-), &{} x<0, \\ u^{(1)}(\eta ;\lambda _1^+,\lambda _2^+,\lambda _3^+), &{} x>0, \end{array}\right. } \quad \text {with}\quad \lambda _1^+=q_-. \end{aligned}$$

The resolution of the step (5.14) is described by

5.15 $$\begin{aligned} \begin{aligned} u(\eta ; x,t) = {\left\{ \begin{array}{ll} u^{(1)}(\eta ;\lambda _1^-,\lambda _2^-,q_+), &{} x<s_-t, \\ u^{(1)}(\eta ;\lambda _1(x/t),\lambda _2^-,q_+), &{}{} s_-t<x<s_+t, \\ u^{(0)}(\eta ;q_+), &{} x>s_+t,\end{array}\right. } \end{aligned} \end{aligned}$$

where $\lambda _3(x/t)$ is given by the $3^+$-wave solution of the modulation equations (4.17):

5.16 $$\begin{aligned} \begin{aligned}&\lambda _1 = q_-,\quad \lambda _2=\lambda _2^+,\quad V_3(\lambda _1,\lambda _2,\lambda _3) = x/t,\quad \text {for}\quad s_-t<x<s_+ t,\\&s_- = V_3(q_-,\lambda _2^+,\lambda _2^+) = 2(q_-)^2+4(\lambda _2^+)^2,\quad s_+ = V_3(q_-,\lambda _2^+,\lambda _3^+). \end{aligned} \end{aligned}$$

**Fig. 6:**
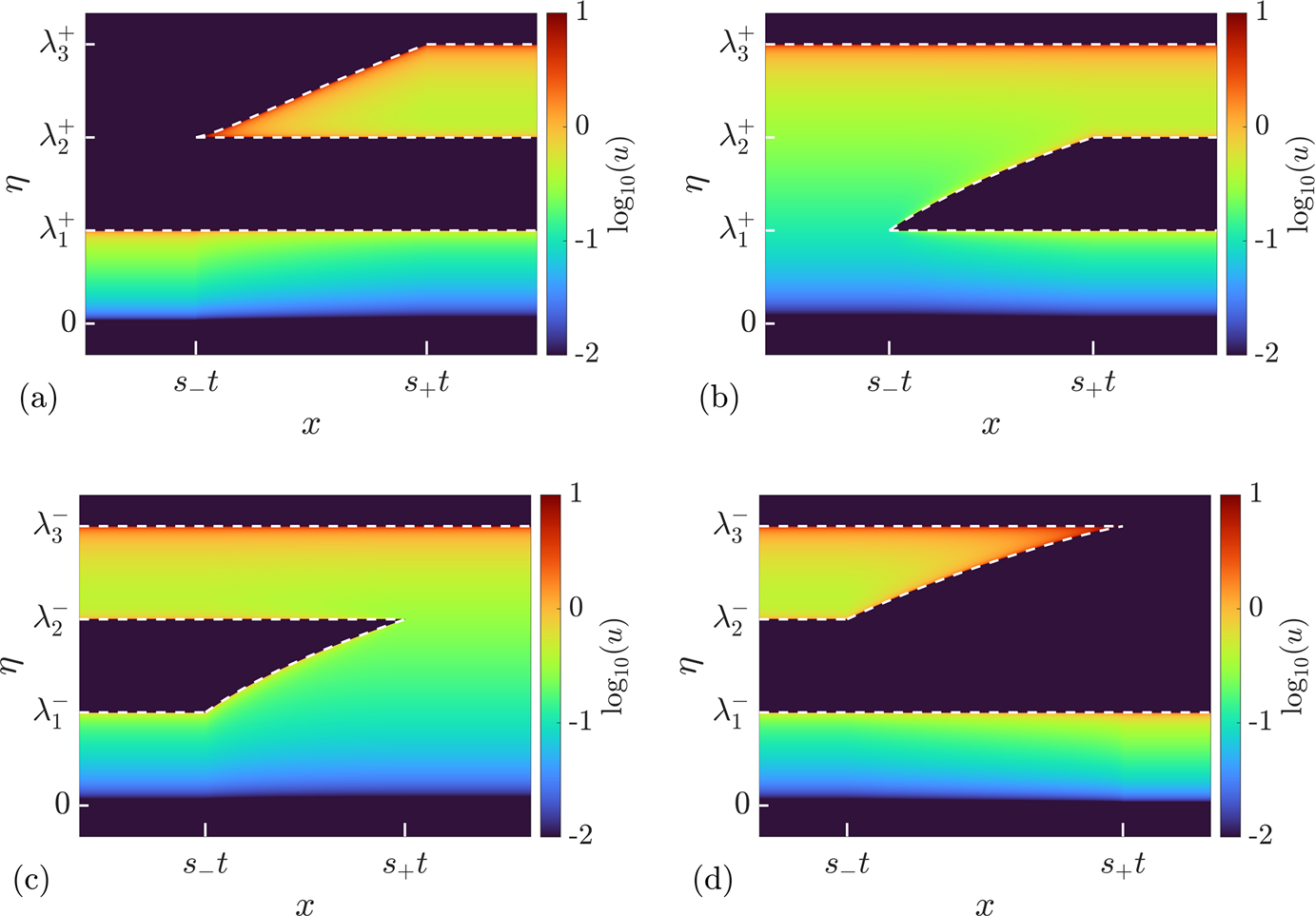
Basic modulation configurations in the Riemann problem (1.1), (5.1) for soliton condensates with $N_-+N_+=1$. **a** $3^+$-wave solution (5.16). **b** $2^+$-wave solution (5.19). **c** $1^-$-wave solution (5.22). **d** $2^-$-wave solution (5.25). In all cases, the dashed lines show the variation of the spectral edges $\lambda _1\le \lambda _2 \le \lambda _3$, and the colors visualize the DOS $u^{(1)}(\eta ; {\varvec{\lambda }})$ (Color figure online)

The behavior of the Riemann invariants $\lambda _j$ in the $3^+$-wave is shown in Fig. 6a. The associated soliton condensate KdV solution $\varphi (x,t)$ along with the behavior of the mean $\langle \varphi \rangle$ is shown in Figs. 10 and 11.

(ii) $$2^+$$-*wave*

Consider the initial condition:

5.17 $$\begin{aligned} u(\eta ; x,t=0) = {\left\{ \begin{array}{ll} u^{(0)}(\eta ;q_-), &{} x<0, \\ u^{(1)}(\eta ;\lambda _1^+,\lambda _2^+,\lambda _3^+), &{} x>0, \end{array}\right. } \quad \text {with}\quad \lambda _3^+=q_-. \end{aligned}$$

The resolution of the step (5.17) is described by:

5.18 $$\begin{aligned} u(\eta ; x,t) = {\left\{ \begin{array}{ll} u^{(0)}(\eta ;q_-), &{} x<s_-t, \\ u^{(1)}(\eta ;\lambda _1^+,\lambda _2(x/t),q_-), &{} s_-t<x<s_+t, \\ u^{(1)}(\eta ;\lambda _1^+,\lambda _2^+,q_-), &{} x>s_+t,\end{array}\right. } \end{aligned}$$

where $\lambda _2(x/t)$ is given by the $2^+$-wave solution of the modulation equations (4.17):

5.19 $$\begin{aligned} \begin{aligned}&\lambda _1 = \lambda _1^+,\quad V_2(\lambda _1,\lambda _2,\lambda _3) = x/t,\quad \lambda _3=q_-,\quad \text {for}\quad s_-t<x<s_+ t,\\&s_- = V_2(\lambda _1^+,\lambda _1^+,\lambda _2^+) = 12(\lambda _1^+)^2-6(q_-)^2,\quad s_+ = V_2(\lambda _1^+,\lambda _2^+,q_-). \end{aligned} \end{aligned}$$

The behavior of the Riemann invariants $\lambda _j$ in the $2^+$-wave is shown in Fig. 6b.

(iii) $$1^-$$-*wave*

Consider the initial condition:

5.20 $$\begin{aligned} u(\eta ; x,t=0) = {\left\{ \begin{array}{ll} u^{(1)}(\eta ;\lambda _1^-,\lambda _2^-,\lambda _3^-), &{} x>0, \\ u^{(0)}(\eta ;q_+), &{} x<0, \end{array}\right. } \quad \text {with}\quad \lambda _3^-=q_+. \end{aligned}$$

The resolution of the step (5.20) is described by

5.21 $$\begin{aligned} u(\eta ; x,t) = {\left\{ \begin{array}{ll} u^{(1)}(\eta ;\lambda _1^-,\lambda _2^-,q_+), &{} x<s_-t, \\ u^{(1)}(\eta ;\lambda _1(x/t),\lambda _2^-,q_+), &{} s_-t<x<s_+t, \\ u^{(0)}(\eta ;q_+), &{} x>s_+t,\end{array}\right. } \end{aligned}$$

where $\lambda _1(x/t)$ is given by the $1^-$-wave solution of the modulation equations (4.17):

5.22 $$\begin{aligned} \begin{aligned}&V_1(\lambda _1,\lambda _2,\lambda _3) = x/t,\quad \lambda _2 = \lambda _2^-,\quad \lambda _3=q_+,\quad \text {for}\quad s_-t<x<s_+ t,\\&s_- = V_1(\lambda _1^-,\lambda _2^-,q_+),\quad s_+ = V_1(\lambda _2^-,\lambda _2^-,q_+) = 12(\lambda _2^-)^2-6(q_+)^2. \end{aligned} \end{aligned}$$

The behavior of the Riemann invariants $\lambda _j$ in the $1^-$-wave is shown in Fig. 6c.

(iv) $$2^-$$-*wave*

Consider the initial condition:

5.23 $$\begin{aligned} u(\eta ; x,t=0) = {\left\{ \begin{array}{ll} u^{(1)}(\eta ;\lambda _1^-,\lambda _2^-,\lambda _3^-), &{} x<0, \\ u^{(0)}(\eta ;q_+), &{} x>0, \end{array}\right. } \quad \text {with}\quad \lambda _1^-=q_+. \end{aligned}$$

The resolution of the step (5.23) is described by

5.24 $$\begin{aligned} u(\eta ; x,t) = {\left\{ \begin{array}{ll} u^{(1)}(\eta ;q_+,\lambda _2^-,\lambda _3^-), &{} x<s_-t, \\ u^{(1)}(\eta ;q_+,\lambda _2(x/t),\lambda _3^-), &{} s_-t<x<s_+t, \\ u^{(0)}(\eta ;q_+), &{} x>s_+t,\end{array}\right. } \end{aligned}$$

where $\lambda _2(x/t)$ is given by the $2^-$-wave solution of the modulation equations (4.17):

5.25 $$\begin{aligned} \begin{aligned}&\lambda _1 = q_+,\quad V_2(\lambda _1,\lambda _2,\lambda _3) = x/t,\quad \lambda _3=\lambda _3^-,\quad \text {for}\quad s_-t<x<s_+ t,\\&s_- = V_2(q_+,\lambda _2^-,\lambda _3^-),\quad s_+ = V_2(q_+,\lambda _3^-,\lambda _3^-)= 2(q_+)^2+4(\lambda _3^-)^2. \end{aligned} \end{aligned}$$

The behavior of the Riemann invariants $\lambda _j$ in the $2^-$-wave is shown in Fig. 6d. The associated soliton condensate KdV solution $\varphi (x,t)$ along with the behavior of the mean $\langle \varphi \rangle$ is shown in Figs. 12 and 13.

## Riemann Problem: Numerical Results

We consider Riemann problems with $N_-+N_+ \le 1$. Because of the inherent limitations of the numerical implementation of soliton gas detailed in Appendix A, we restrict the comparison to the cases $q_-=0$ or $q_+=0$.

### Rarefaction Wave

In this first example, we choose

6.1 $$\begin{aligned} \{N; {\varvec{\lambda }}\}( x,t=0) = {\left\{ \begin{array}{ll} \{0; q_-=0 \}, &{} x<0, \\ \{0; q_+=1 \}, &{} x>0. \end{array}\right. } \end{aligned}$$

**Fig. 7:**
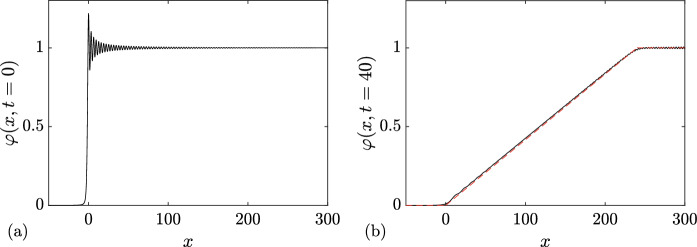
Riemann problem with initial condition (6.1) for DOS $u(\eta ; x,t)$. The plots depict the variation of a condensate’s realization $\varphi (x,t)$ at $t=0$ (**a**) and $t=40$ (**b**, solid line). The red dashed line depicts the variation of the rarefaction wave $\varphi = \lambda _1(x/t)^2$ (5.6) (Color figure online)

**Fig. 8:**
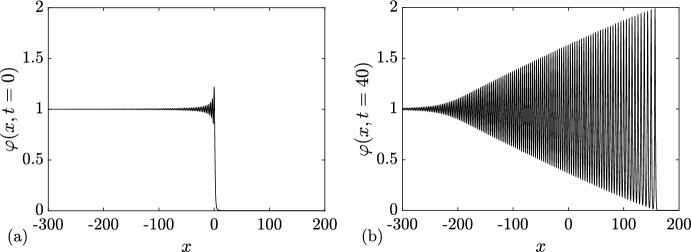
Riemann problem with initial condition (6.2) for DOS $u(\eta ; x,t)$. The plots depict the variation of a condensate’s realization $\varphi (x,t)$ at $t=0$ (**a**) and $t=40$ (**b**)

A numerical realization of the soliton condensate evolution corresponding to the steplike initial condition (6.1) is displayed in Fig. 7. The same figure displays the realization at $t=40$. The realization corresponds to a *n*-soliton solution with parameters distributed according to the initial DOS of (5.3), (6.1); details are given in Appendix A. As predicted in Sect. 4.1, the realization of the condensate corresponds to the vacuum $\varphi =0$ at the left of $x=0$, and a constant $\varphi =1$ at the right of $x=0$. As highlighted in Appendix A.1, the *n*-soliton solution displays an overshoot at $x=0$, regardless of the number of solitons *n*, which is reminiscent of Gibbs’ phenomenon in the theory of Fourier series. This phenomenon has been originally observed in the numerical approximation of the soliton condensate of the focusing NLS equation by a *n*-soliton solution in Gelash et al. (2021); see, for instance, the similarities between Figs. 7a, 8a and Fig. 2a of Gelash et al. (2021). Indeed, in both cases, the IST spectrum of the step distribution contains a non-solitonic radiative component (cf. Ablowitz 2011), which is not taken into account by the *n*-soliton solution; the mismatch between the exact step and the *n*-soliton solution manifests by the occurrence of the spurious oscillations observed near $x=0$.

The solution of the Riemann problem with the initial condition (6.1) is given by $u^{(0)}(\eta ; \lambda _1(x,t))$ where $\lambda _1(x,t)$ is the rarefaction wave (genus 0) solution (5.6). We have shown in Sect. 4.1 that the genus 0 soliton condensate is almost surely described by the constant solution $\varphi = (\lambda _1)^2$. In the context of the evolution of the step (6.1) $\lambda _1$ varies according to (5.6) so $\lambda (x,t)$ should be treated as a slowly varying (locally constant) condensate solution. In Fig. 7, we compare the numerical realization of the evolution of genus 0 condensate with the analytical solution (5.6).

### Dispersive Shock Wave

We now consider

6.2 $$\begin{aligned} \{N; {\varvec{\lambda }}\}( x,t=0) = {\left\{ \begin{array}{ll} \{0; q_-=1 \}, &{} x<0, \\ \{0; q_+=0 \}, &{} x>0. \end{array}\right. } \end{aligned}$$

A numerical realization of the genus 0 soliton condensate corresponding to the step-initial condition (6.2) is presented in Fig. 8a: it corresponds to the vacuum $\varphi =0$ for $x>0$, and a constant $\varphi =1$ for $x<0$. The realization at $t=40$ is shown in Fig. 8b, and it corresponds to a classical DSW solution for the KdV equation.

The solution of the condensate Riemann problem with the initial condition (5.3), (6.2) is given by the genus 1 DOS (5.9) modulated by the 2-wave solution (5.10) of the Whitham equations. In order to make a quantitative comparison of this analytical solution with the numerical evolution of the soliton gas displayed in Fig. 8, we compute numerically the mean $\langle \varphi \rangle$ and the variance $\sqrt{\langle \varphi ^2 \rangle - \langle \varphi \rangle ^2}$, the latter being an amplitude type characteristic of the cnoidal wave. We have conjectured in Sect. 4.1 that any realization of the uniform genus 1 condensate corresponds to a cnoidal wave modulo the initial phase $\theta ^0\in [0;2\pi )$. In that case, the ensemble average of the soliton condensate reduces to an average over the phase $\theta ^0$, or equivalently, over the period of the cnoidal wave, which can be performed on a single realization. We assume here that the result generalizes to non-uniform condensates so that the realization computed numerically and displayed in Fig. 8b can be consistently compared with a slowly modulated cnoidal wave solution. The averages $\langle \varphi (x,t) \rangle$ and $\langle \varphi (x,t)^2 \rangle$ can be determined via a local phase average of one realization of the condensate. The local period averages are obtained via

**Fig. 9:**
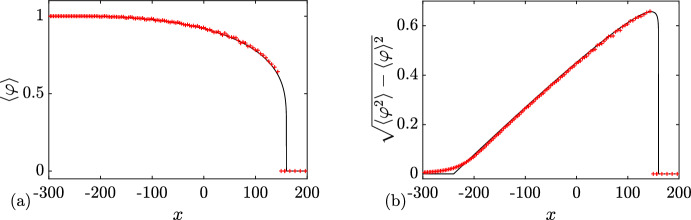
Mean $\langle \varphi \rangle$ (**a**) and variance $\sqrt{\langle \varphi ^2 \rangle - \langle \varphi \rangle ^2}$ (**b**) of the solution of the Riemann problem’s solution with the initial condition (6.2). The markers correspond to averages extracted from the numerical solution using (6.3), and the solid black lines to the corresponding analytical averages (4.13), (4.14) evaluated on the Gurevich–Pitaevskii modulation solution (5.10)

6.3 $$\begin{aligned} \begin{aligned} \langle \varphi (x,t) \rangle&= \frac{1}{L(x,t)} \int _x^{x+L(x,t)} \varphi (y,t) \textrm{d}y, \\ \langle \varphi (x,t)^2 \rangle&= \frac{1}{L(x,t)} \int _x^{x+L(x,t)} \varphi (y,t)^2 \textrm{d}y, \end{aligned} \end{aligned}$$

where *L*(*x*, *t*) is the local wavelength extracted numerically. In the examples displayed in Figs. 9, 11, 13, the values of $\langle \varphi (x,t) \rangle$ and $\langle \varphi (x,t)^2 \rangle$ are computed at each maximum $x=x_i$ of the realization $\varphi (x,t)$, and the local wavelength is given by $L(x_i,t)=(x_{i+1}-x_{i-1})/2$.

The comparison between the analytically determined averages (4.13), (4.14) evaluated on the Gurevich–Pitaevskii modulation solution (5.10) for DSW and the averages (6.3) obtained numerically is presented in Fig. 9 and shows a very good agreement.

### Generalized Rarefaction Wave

$N_-+N_+=0$ in the two previous examples. In the next examples, we choose $N_-+N_+=1$. Let’s start with $N_+=1$:

6.4 $$\begin{aligned} \{N; {\varvec{\lambda }}\}( x,t=0) = {\left\{ \begin{array}{ll} \{0; q_-=0 \}, &{} x<0, \\ \{1; (\lambda _1^+=0, \lambda _2^+=1/2,\lambda _2^+=1)\}, &{} x>0. \end{array}\right. } \end{aligned}$$

**Fig. 10:**
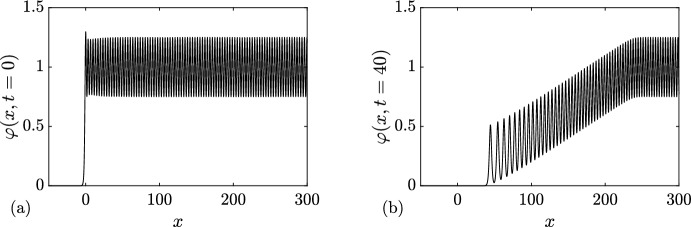
Riemann problem for soliton condensate with initial condition (6.4) for DOS $u(\eta ; x,t)$. The plots depict the variation of a condensate’s realization $\varphi (x,t)$ at $t=0$ (**a**) and $t=40$ (**b**)

A numerical realization of the step-initial condition is displayed in Fig. 10. The same figure displays the realization at $t=40$. The realization of the condensate corresponds to the “vacuum” $\varphi =0$ for $x<0$, and a cnoidal wave for $x>0$. Note that the KdV equation does not admit heteroclinic traveling wave solutions, rendering difficult the numerical implementation of these “generalized” Riemann problems studied, for instance, in Sprenger and Hoefer (2020); Gavrilyuk et al. (2020). Remarkably here, the solution depicted in Fig. 10 is an exact, *n*-soliton solution of the KdV equation. As highlighted previously (see also Appendix A.1), the *n*-soliton solution exhibits an overshoot at $x=0$, regardless of the number of solitons *n*.

**Fig. 11:**
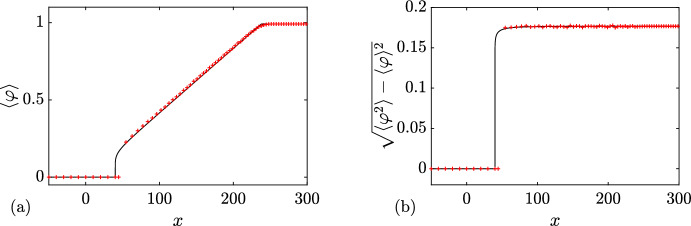
Mean $\langle \varphi \rangle$ (**a**) and variance $\sqrt{\langle \varphi ^2 \rangle - \langle \varphi \rangle ^2}$ (**b**) of the solution of the Riemann problem’s solution with the initial condition (6.4). The markers correspond to the averages extracted from the numerical solution using (6.3), and the solid black lines to the corresponding analytical averages (4.13), (4.14), (5.16)

The solution of the Riemann problem for the kinetic equation with the initial condition (5.14), (6.4) is given by the $3^+$-wave (5.15), (5.16). The comparison between the analytical averages (4.13), (4.14), (5.16) and the averages obtained numerically is shown in Fig. 11 and shows a very good agreement. The modulation depicted in Figs. 10b and 11a resembles the modulation of a cnoidal wave of an almost constant amplitude but with a varying mean. The variation of the mean $\langle \varphi \rangle$ is similar to the variation of the field in a classical rarefaction wave, so we call the corresponding structure shown in Fig. 10b a *generalized rarefaction wave*. The variance of the wavefield $\varphi$ in the generalized rarefaction wave is shown in Fig. 11b.

### Generalized Dispersive Shock Wave

We now consider the “complementary” initial condition

6.5 $$\begin{aligned} \{N; {\varvec{\lambda }}\}( x,t=0) = {\left\{ \begin{array}{ll} \{1; (\lambda _1^-=0, \lambda _2^-=1/2,\lambda _2^-=1)\}, &{} x<0, \\ \{0; q_+=0 \}, &{} x>0. \end{array}\right. } \end{aligned}$$

**Fig. 12:**
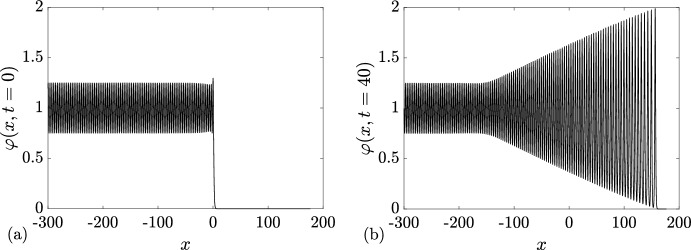
Riemann problem with initial condition (6.5) for DOS $u(\eta ; x,t)$. The plots depict the variation of a condensate’s realization $\varphi (x,t)$ at $t=0$ (**a**) and $t=40$ (**b**)

An example of the numerical realization of the soliton gas step-initial condition and its evolution at $t=40$ are displayed in Fig. 12.

**Fig. 13:**
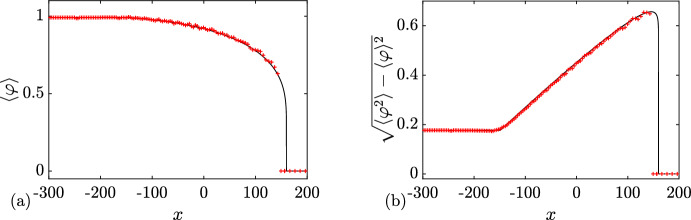
Mean $\langle \varphi \rangle$ (**a**) and variance $\sqrt{\langle \varphi ^2 \rangle - \langle \varphi \rangle ^2}$ (**b**) of the solution of the Riemann problem’s solution with the initial condition (6.5). The markers correspond to averages extracted from the numerical solution using (6.3), and the solid black lines to the corresponding analytical averages (4.13), (4.14), (5.16)

The solution of the Riemann problem with the initial condition (6.5) is given by the $2^-$-wave (5.24), (5.25). The comparison between the analytically derived averages (4.13), (4.14), (5.25) and the averages obtained numerically is displayed in Fig. 13 and shows a very good agreement. The modulation observed in Figs. 12, 13 resembles the modulation of partial dispersive shock wave: the modulated cnoidal wave reaches the soliton limit $m=1$ for $x \rightarrow s_+ t$ but terminates at $m \ne 0$ for $x \rightarrow s_-t$. The solution then continues as a non-modulated cnoidal wave for $x < s_- t$. This structure differs from the celebrated dispersive shock wave solution of the KdV equation involving the entire range $0 \le m \le 1$ (El and Hoefer 2016). We call the described structure connecting a constant state (a genus 0 condensate) at $x \rightarrow + \infty$ with a periodic solution (a genus 1 condensate) at $x \rightarrow - \infty$ a *generalized DSW*. We note that the soliton condensate structure shown in Fig. 12b exhibits strong similarity to the “deterministic KdV soliton gas” solution constructed in Girotti et al. (2021).

## Diluted Soliton Condensates

### Equilibrium Properties

We now introduce the notion of a “diluted” soliton condensate by considering DOS $u(\eta )=C u^{(N)} (\eta )$, where $u^{(N)}(\eta )$ is the condensate DOS of genus *N*, and $0<C<1$ is the “dilution constant”.

E.g. the diluted soliton condensate of genus 0 is characterized by DOS

7.1 $$\begin{aligned} u(\eta )= C \frac{\eta }{\pi \sqrt{\lambda _1^2-\eta ^2}}, \quad 0< C < 1. \end{aligned}$$

We recover the genus 0 condensate DOS (4.1) by setting $C=1$. As *C* decreases, the “averaged spacing” between the solitons

**Fig. 14:**
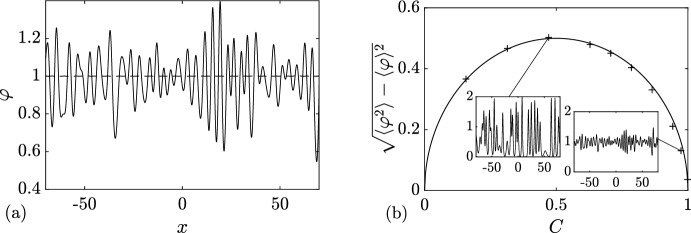
**a** Realizations soliton gas with the DOS (7.1) and $\lambda _1=1$: $C=1$ in dashed line (genus 0 condensate) versus $C= 0.97$ in solid line (diluted genus zero condensate); in both cases, the gas is realized numerically with $N=100$ solitons. **b** Variance for diluted condensates $C<1$. Solid line: formula (7.3); markers: numerically extracted values of the variance; insets: typical realizations of the KdV wave field $\varphi (x,t)$ in diluted condensates

7.2 $$\begin{aligned} \kappa ^{-1} = \left( \int u(\eta ) \textrm{d}\eta \right) ^{-1} \propto C^{-1} \end{aligned}$$

increases and the condensate gets “diluted”. Comparison between the most probable realization of the condensate ($C=1$) and a typical realization of a slightly dilute condensate ($C=0.97$) is given in Fig. 14. Remarkably, one can see that a slight increase of the average spacing between the solitons within the condensate results in the emergence of significant random oscillations of the KdV wave field.

As follows from (2.14), we have $\langle \varphi \rangle = \langle \varphi ^2 \rangle = C$ for the diluted genus 0 condensate so that the variance is given by:

7.3 $$\begin{aligned} \Delta = \sqrt{\langle \varphi ^2 \rangle - \langle \varphi \rangle ^2} = \sqrt{C(1 - C)}. \end{aligned}$$

**Fig. 15:**
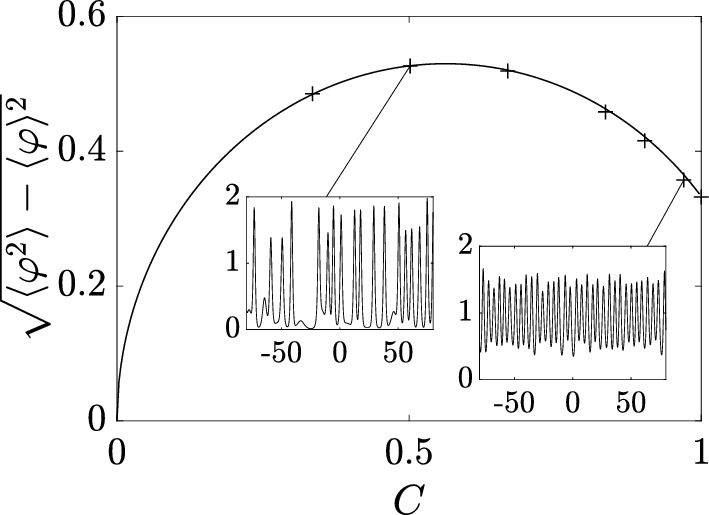
Variance for diluted genus 1 condensates with DOS (7.4) and $(\lambda _1,\lambda _2,\lambda _3)=(0.5,0.85,1)$; insets: typical realizations of the KdV wave field $\varphi (x,t)$ in diluted condensates

The comparison between (7.3) and the variance obtained numerically by averaging over different diluted condensates is presented in Fig. 14. Assuming ergodicity of a generic uniform soliton gas, the ensemble average $\langle \dots \rangle$ in Fig. 14b (and Fig. 15b) is computed here numerically with a spatial average of one, spatially broad, gas realization.

More generally, the diluted soliton condensate of genus *N* is characterized by DOS

7.4 $$\begin{aligned} u(\eta )= C u^{(N)}(\eta ;\lambda _1,\dots ,\lambda _{2N+1}), \quad 0< C < 1. \end{aligned}$$

We have in the general case

7.5 $$\begin{aligned} \langle \varphi \rangle = C \langle \varphi _\textrm{c}^{(N)} \rangle ,\quad \langle \varphi ^2 \rangle = C \langle \big (\varphi _\textrm{c}^{(N)} \big )^2 \rangle , \end{aligned}$$

where $\langle \varphi _\textrm{c}^{(N)} \rangle$, $\langle \big (\varphi _\textrm{c}^{(N)} \big )^2\rangle$ are the ensemble averages obtained for the genuine condensate ($C=1$), which are functions of $\lambda _1,\dots ,\lambda _{2N+1}$ only; for instance, $\langle \varphi _\textrm{c}^{(1)} \rangle$, $\langle \big (\varphi _\textrm{c}^{(1)} \big )^2 \rangle$ are given by (4.13), (4.14). Since $\langle \big (\varphi _\textrm{c}^{(N)} \big )^2\rangle \ne \langle \varphi _\textrm{c}^{(N)} \rangle ^2$ for $N \ge 1$ and distinct $\lambda _i$’s, the variance of diluted genus condensates

7.6 $$\begin{aligned} \Delta = \sqrt{C \langle \big (\varphi _\textrm{c}^{(N)} \big )^2 \rangle - C^2 \langle \varphi _\textrm{c}^{(N)} \rangle ^2} \end{aligned}$$

never vanishes if $N \ge 1$, as can be seen in the example $N=1$ shown in Fig. 15. Thus, in contrast to the genus 0 case, the transition from the genus 1 condensate ($C=1$) to diluted genus 1 condensate ($C<1$) does not see a drastic change in the oscillations’ amplitude. In particular, the oscillations seem to remain “almost” coherent—i.e. an average period can be identified—for the dilution factors *C* close to 1 as depicted in the inset of Fig. 15.

**Fig. 16:**
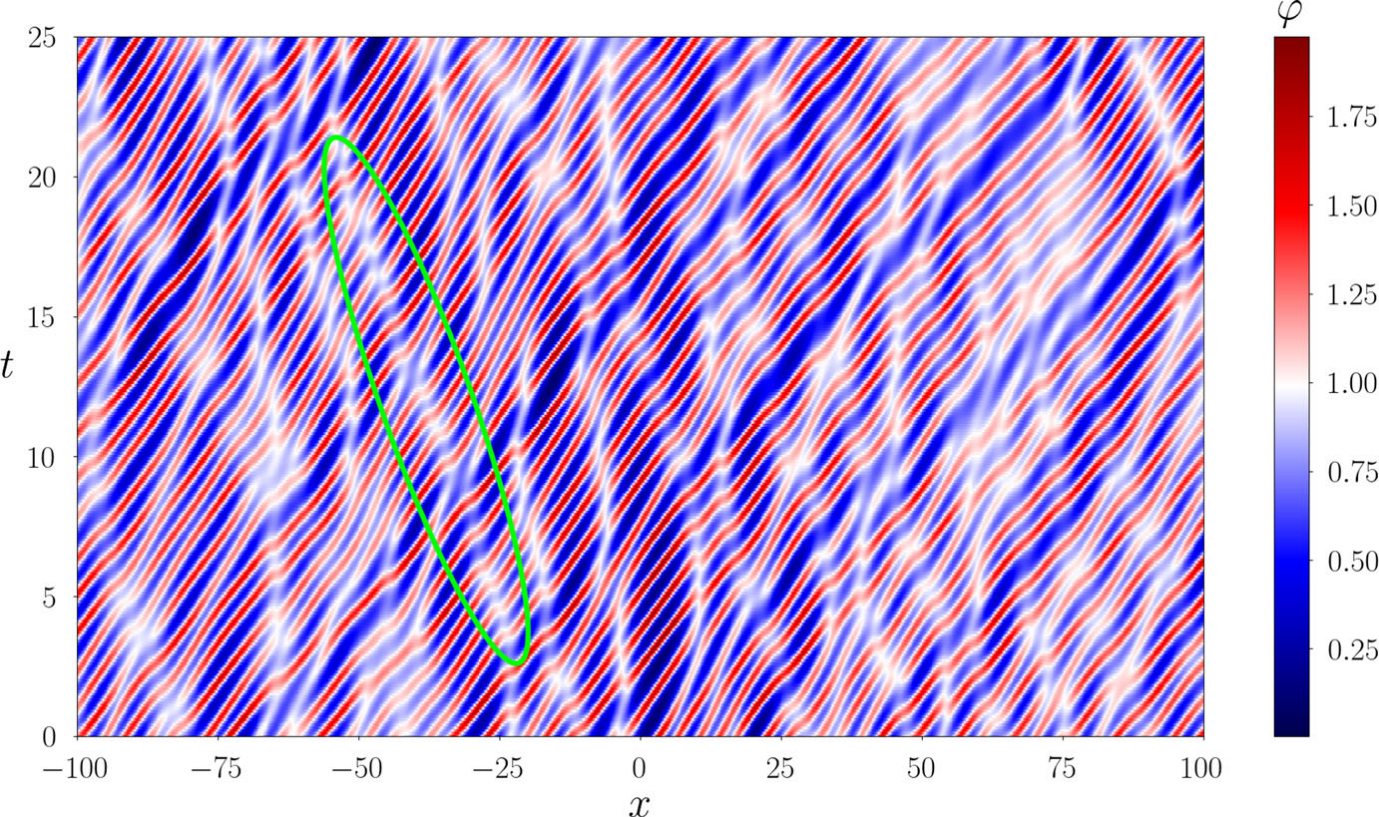
Soliton trajectories in a diluted genus 0 soliton condensate with $C=0.9$. Highlighted is a small-amplitude tracer soliton moving backwards

Diluted condensates provide a convenient framework to verify the prediction formulated in Remark 2.1 regarding the “backflow” effect (i.e. the existence of tracer KdV solitons moving in negative direction) in sufficiently dense soliton gases. A numerical simulation of the diluted genus 0 condensate with $C=0.9$ where one can clearly see the soliton trajectory with a negative slope is presented in Fig. 16.

### Riemann Problem

We can now consider the Riemann problem for diluted condensates for which the initial DOS (5.1) is replaced by

7.7 $$\begin{aligned} u(\eta ; x,t=0) = {\left\{ \begin{array}{ll} C_- \,u^{(N_-)}(\eta ;\lambda _1^-, \dots , \lambda _{2N_-+1}^-), &{} x<0, \\ C_+ \,u^{(N_+)}(\eta ;\lambda _1^+, \dots , \lambda _{2N_++1}^+), &{} x>0, \end{array}\right. } \end{aligned}$$

where $$0<C_\pm <1$$.

**Fig. 17:**
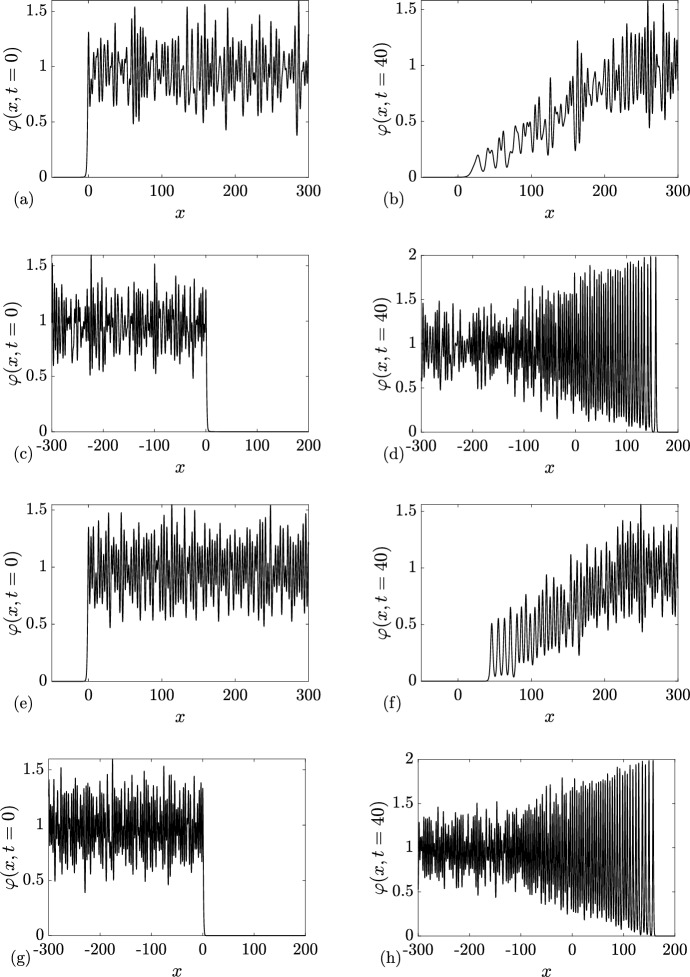
Riemann problem for diluted soliton condensates with the initial condition (7.7) with $C_\pm =0.95$. **a**–**d** $N_-+N_+=0$ and $\lambda _1=1$; **e**–**h** $N_-+N_+=1$ and $(\lambda _1,\lambda _2,\lambda _3)=(0,1/2,1)$. The diluted condensates are realized with exact *n*-soliton solutions ($n=200$) configured spectrally according to the respective scaled condensate DOSs. The evolution results in the generation of incoherent rarefaction and dispersive shock waves

To be specific, we investigate numerically the evolution of the diluted condensate initial conditions (7.7) with $N_-+N_+\le 1$ and $\lambda _i$ chosen from the examples presented in Sect. 6. Numerical realizations of the step-initial condition and their evolution in time are presented in Fig. 17. One can see that generally, realizations of the diluted soliton condensate do not exhibit a macroscopically coherent structure as observed in Sect. 6. However, in the case $N_-+N_+=1$, the evolution of the diluted condensate realizations, despite the visible incoherence, still qualitatively resembles the evolution of the “genuine” condensates depicted in Figs. 10, 12. One can see that the recognizable patterns of the generalized rarefaction wave (see Fig. 17f) and the generalized DSW (see Fig. 17h) persist even if $C < 1$. Indeed, as shown in Sect. 7.1, the oscillations in a realization of the diluted genus 1 condensate appear almost coherent for a small dilution factor. The persistence of coherence can also be observed in the case $N_-+N_+=0$ when $\lambda _1^- > \lambda _1^+$ (Fig. 17d): a DSW develops if $C=1$, and coherent, finite amplitude oscillations still develop for $C\ne 1$ at the right edge of the structure where the amplitudes of oscillations are large. In connection with the above, it is important to note that, although the initial condition (7.7) is given by a discontinuous diluted condensate DOS, $u(\eta ; x,0)=Cu^{(N)}(\eta )$, the kinetic equation evolution does not imply that the DOS will remain to be of the same form for $t>0$. In other words, unlike genuine condensates, the diluted condensates do not retain the spectral “diluted condensate” property during the evolution.

## Conclusions and Outlook

We have considered a special class of soliton gases for the KdV equation, termed soliton condensates, which are defined by the property of vanishing the spectral scaling function $\sigma (\eta )$ in the soliton gas nonlinear dispersion relations (2.9), (2.10). As a result, the DOS $u(\eta )$ in a soliton condensate is uniquely determined by its spectral support $\Gamma ^+ \in {\mathbb {R}}^+$. By considering $\Gamma ^+$ to be a union of $N+1$ disjoint intervals, $[0, \lambda _1]\cup [\lambda _2, \lambda _3] \cup \dots \cup [\lambda _{2N}, \lambda _{2N+1}]$, and allowing the endpoints $\{\lambda _j\}_{j=1}^{2N+1}$ vary slowly in space-time we prove that the kinetic equation for soliton gas reduces in the condensate limit to the genus *N* KdV–Whitham modulation for $\lambda _j(x,t)$. The KdV–Whitham equations were originally derived via the wave averaging procedure in Whitham (1965), Flaschka et al. (1980) and via the semiclassical limit of the KdV equation in Lax and Levermore (1983). These equations have been extensively used for the description of dispersive shock waves (El and Hoefer 2016), particularly in the context of dispersive Riemann problem originally introduced by Gurevich and Pitaevskii (1974). One important implication of this reduction of the soliton gas kinetics to the modulation dynamics of finite gap potentials is the invariance of the definitive condensate property $\sigma =0$ with respect to the time evolution, i.e. soliton condensate remains a condensate during the evolution; however, its genus *N* can change (increase) as a result of the wavebreaking of the multivalued spectral curve $\Lambda _N(x,t) \equiv \{\lambda _1(x,t), \lambda _2(x,t), \dots , \lambda _{2N+1}(x,t) \}$. We note that the genuine nonlinearity of the Whitham modulation equations describing the evolution of soliton condensates is in sharp contrast to linear degeneracy of the multicomponent “cold-gas”hydrodynamic reductions of the kinetic equation studied earlier (El et al. 2011). We also mention intriguing parallels between the modulation dynamics of soliton condensates and the hydrodynamic properties of the so-called zero-entropy states in generalized hydrodynamics studied in Doyon et al. (2017).

Along with the characterization of the large-scale, modulation, dynamics of soliton condensates, our work suggests that they represent “coherent” or “deterministic” soliton gases whose typical realizations are given by finite-gap potentials. We prove this conjecture for genus 0 condensates and present strong numerical evidence for $N=1,2$.

By invoking the results from the modulation theory of dispersive shock waves, we have constructed analytical solutions to several Riemann problems for the soliton gas kinetic equation subject to discontinuous condensate initial data. These solutions describe the evolution of generalized rarefaction and dispersive shock waves in soliton condensates. We performed numerical simulations of the Riemann problem for the KdV soliton condensates by constructing an exact *n*-soliton solutions with *n* large and the spectral parameters distributed according to the condensate DOS. A comparison of the numerical simulations with analytical predictions from the solutions of the kinetic equation showed excellent agreement. Finally, we considered the basic properties of “diluted” soliton condensates with a scaled condensate DOS, exhibiting rich incoherent behaviors.

There are several avenues for future work suggested by our results. One natural direction is the extension of the developed KdV soliton condensate theory to other integrable equations. Such an extension looks pretty straightforward for the defocusing NLS and other equations whose finite-gap solutions are associated with self-adjoint Lax operators (defocusing mKdV, Kaup-Boussinesq, etc.) and exhibit close similarities to the KdV case. There is a rich literature on the corresponding Whitham equations and their solutions describing the modulations in the rarefaction and dispersive shock waves (see, e.g. El and Hoefer 2016 and references therein). These results can now be naturally re-interpreted within the soliton gas context with potentially important implications for water waves, nonlinear optics and condensed matter physics. A related challenging problem of major interest is non-equilibrium dynamics of soliton condensates for the focusing NLS equation. Unlike the KdV soliton condensates, their focusing NLS counterparts exhibit strong incoherence and were shown to provide an accurate description of the long-time development of spontaneous modulational instability (Gelash et al. 2019). There are a growing number of theoretical and experimental research on soliton and breather gases for the focusing NLS equation (see El and Tovbis 2020; Roberti et al. 2021; Gelash et al. 2021; Bertola et al. 2023) posing further intriguing questions pertinent to the realm of integrable turbulence (Zakharov 2009).

Yet another interesting problem to be considered is the near-condensate soliton gas dynamics realized by assuming the spectral scaling function $\sigma$ to be “small” ($\sigma (\eta ) = \epsilon {\tilde{\sigma }} (\eta )$, $\epsilon \ll 1$, ${\tilde{\sigma }} = {\mathcal {O}}(1)$). And last but not least, one of the most intriguing open questions is the possibility of phase transitions in soliton gases, i.e. the formation of a soliton condensate from non-condensate initial data. The generalized hydrodynamics approach to the thermodynamics of soliton gases (Bonnemain et al. 2022) provides a promising framework to explore this possibility. At the same time, this direction of research could require some departure from integrability and the development of the soliton gas theory for perturbed integrable equations.

## Figures and Tables

**Fig. 18:**
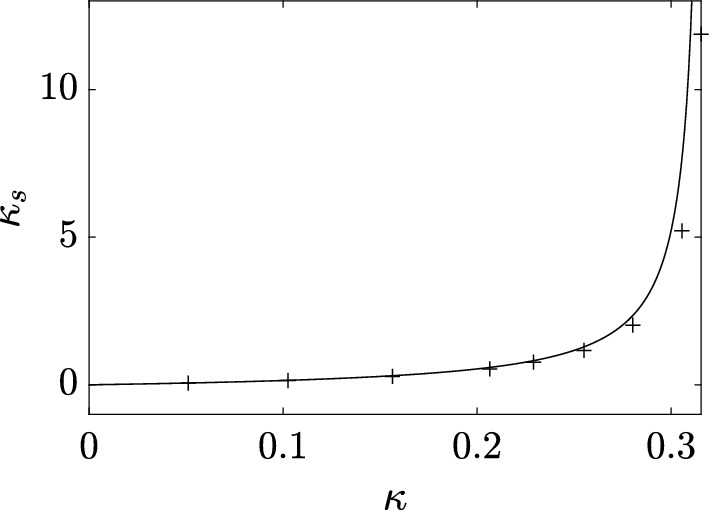
The solid line represents the variation of $\kappa _s$ with respect to $\kappa$ for a diluted genus 0 condensate, cf. (A.7). The markers are obtained using the 100-soliton solution: $\kappa =\ell /n$ where $\ell$ corresponds to the spatial extension of the *n*-soliton solution

**Fig. 19:**
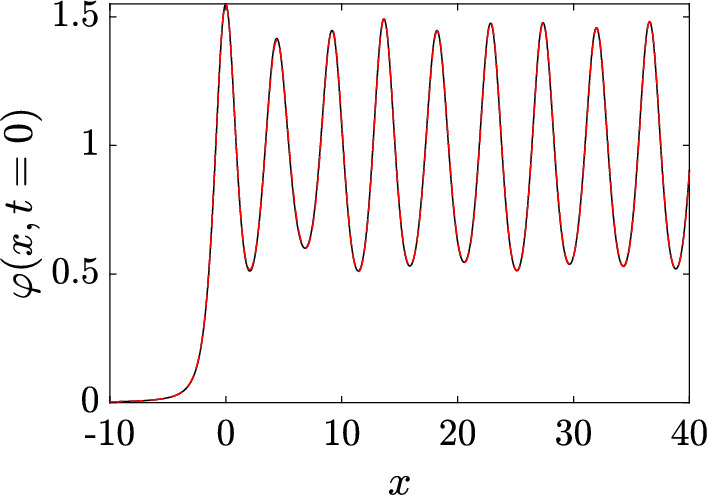
*n*-Soliton solution approximating a realization of the condensate $(N; \lambda _1,\lambda _2,\lambda _3) = (1; 0,0.5,0.85)$. The solid black line represents the solution $n=100$, and the red dashed line the solution $n=200$. Both solutions have been shifted such that the maximum of the solution is located at $x=0$ (Color figure online)

**Fig. 20:**
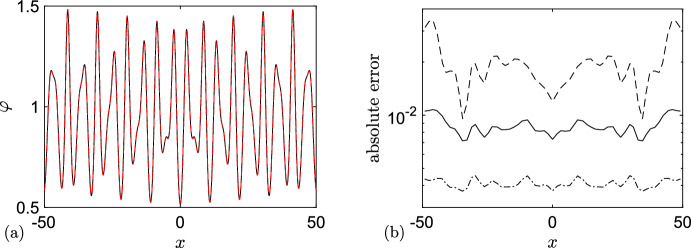
**a** Comparison between KdV genus 2 soliton condensate realized via $n=204$-soliton solution (solid line) and the exact 2-phase KdV solution (dashed line) for $\lambda _1=0.3,\lambda _2=0.5,\lambda _3=0.7,\lambda _4=0.9,\lambda _5=1.$; the two plots are visually indistinguishable from one another; **b** Absolute error for the condensate generated with 50 solitons (dashed line), 100 solitons (solid line) and 204 solitons (dash-dotted line); for readability the absolute error is evaluated at the extrema of the solutions (Color figure online)

## Data Availability

All data analyzed during this study are included in this article.

## Numerical Implementation of Soliton Gas

### Riemann Problem

The realizations of the soliton gas are approximated numerically by the *n*-soliton solution

A.1 $$\begin{aligned} \varphi \equiv \varphi _n(x,t;\eta _1,\dots ,\eta _n,x^0_1,\dots ,x^0_n),\quad n \in {\mathbb {N}}, \end{aligned}$$

where the $\eta _i$’s and $x^0_i$’s correspond, respectively, to the spectral parameters and the “spatial phases” of the solitons; $\eta _i<\eta _{i+1}$ by convention. The numerical implementation of (A.1) is described in Sect. A.3 below. The numerical solutions presented in this work are all generated with $n=200$ solitons, unless otherwise stated.

Since *n* is finite, the *n*-soliton solution reduces to a sum of separated solitons in the limit $|t| \rightarrow \infty$. By construction, we have in the limit $t\rightarrow \pm \infty$

A.2 $$\begin{aligned} \varphi _n(x,t) \sim \sum _{i=1}^n 2\eta _i^2 \textrm{sech}^2 \left[ \eta _i(x - 4 \eta _i^2 t - x_i^\pm ) \right] , \end{aligned}$$

where $x_i^\pm$ are the spatial phases of the *i*-th soliton at $t \rightarrow \pm \infty$. We then take the spatial phase in (A.1) to be $x_i^0 = (x_i^-+x_i^+)/2$.

Consider a uniform soliton gas with the DOS $u(\eta )$. Let the spectral parameters $\eta _i$ be distributed on $\Gamma ^+$ with density

A.3 $$\begin{aligned} \phi (\eta ) = \frac{u(\eta )}{\kappa },\quad \kappa = \int _{\Gamma _+} u(\eta ) \textrm{d} \eta , \end{aligned}$$

where the normalization by the spatial density of solitons $\kappa$ ensures that $\phi (\eta )$ is normalized to 1. It was shown in Gurevich et al. (2000) that the spatial density $\kappa$ is obtained if the phases $x^0_i$ are uniformly distributed on the interval

A.4 $$\begin{aligned} I_s = \left[ -\frac{n}{2\kappa _s},+\frac{n}{2\kappa _s} \right] ,\quad \kappa _s = \int _{\Gamma _+ } \frac{\eta }{\sigma (\eta )} \textrm{d} \eta , \end{aligned}$$

where $\sigma (\eta )$ is the spectral scaling function in the NDRs (2.9), (2.10) ($y(\eta )$ in Gurevich et al. (2000) is given here by $y(\eta ) = u(\eta ) \sigma (\eta )/\eta$). The derivation of (A.4) has been revisited recently in the context of generalized hydrodynamics (Bonnemain et al. 2022): it was shown that $\kappa _s$ corresponds to the density of spatial phases $x_i^0$, or equivalently $x_i^\pm$ which are well defined asymptotically ($t \rightarrow \pm \infty$) where the solitons are “non-interacting” and their position is given by $x_i(t) \sim 4\eta _i^2 t + x_i^\pm$. In the rarefied gas limit, the interaction term in the NDR (2.9) is small and therefore $\sigma (\eta )u(\eta ) \approx \eta$ so that we obtain $\kappa _s = \kappa$ as expected. In the general case, though the density $\kappa _s$ of non-interacting phases is different from the “physical” density $\kappa$, as demonstrated with the soliton condensate examples below. In the thermodynamic limit $n \rightarrow \infty$, the soliton solution (A.1) represents a realization of the uniform soliton gas.

Since the number *n* of solitons is finite, the *n*-soliton solution has a finite spatial extent. By distributing the phases $x^0_i$ uniformly on the interval $I_s$, the *n*-soliton solution $\varphi _n(x,t=0)$ approximates a realization of the uniform soliton gas for $x \in [-\ell /2,\ell /2]$ where $\ell =n/\kappa$; $\varphi _n(x,t=0) \sim 0$ outside of this interval. This naturally generates the box-like initial condition for the kinetic equation

A.5 $$\begin{aligned} u(\eta ;x,t=0) \sim {\left\{ \begin{array}{ll} 0, &{} x<-\ell /2, \\ u(\eta ), &{} -\ell /2<x<\ell /2, \\ 0, &{} \ell /2<x. \end{array}\right. } \end{aligned}$$

Note that $u(\eta ;x,t=0) = 0$ can be seen as the genus 0 condensate where the end point of the central s-band $\lambda _1 \rightarrow 0$. This limits the type of initial condition that can be implemented for the Riemann problem and we choose in practice $(N_-=0,q_-=0)$ or $(N_+=0,q_+=0)$. For convenience, we shift the *x*-axis by $\pm \ell /2$ to obtain one of the discontinuities at the position $x=0$.

The evolution in time of the soliton gas realization is obtained by varying the parameter *t*. Contrarily to a direct resolution of the KdV equation, via finite difference or spectral method, the time-evolution presented here is instantaneous and does not accumulate any numerical errors since the *n*-soliton solution is an exact solution. For the Riemann problem, the maximal time is bounded by the finite extent of the *n*-soliton solution: after a sufficiently long time, the two hydrodynamic states originating from the discontinuities at $x=-\ell /2$ and $x=\ell /2$ start interacting. Longer times can be reached by choosing a larger number of solitons *n*.

We consider now the DOS of interest for this work:

A.6 $$\begin{aligned} u(\eta ) = C u^{(N)}(\eta ; \lambda _1, \dots \lambda _{2N+1}),\quad C\le 1, \end{aligned}$$

where $u^{(N)}$ is DOS of the genus *N* condensate defined in Sect. 3. (A.4) rewrites

A.7 $$\begin{aligned} \kappa _s = \frac{\kappa (C)}{1-C},\quad \kappa (C) = C \int _{\Gamma _+} u^{(N)}(\eta ; \lambda _1, \dots , \lambda _{2N+1}) \textrm{d} \eta . \end{aligned}$$

Figure 18 shows the comparison between the spatial density of solitons $\kappa$ and the density of phases $\kappa _s$ for the genus 0 case where $\kappa (C) = C/\pi$. The phases density $\kappa _s$ diverges in the condensate limit $C\rightarrow 1$, and $x_i^0$’s are all equal to the same phase $x^0$ ($I_s \rightarrow \{x^0\}$). This limit is in agreement with the results obtained in Sect. 4.1 for genus 0 and genus 1 condensates: each realization of the condensate ($C=1$) is approximated by a *coherent* *n*-soliton solution where $x_i^0=x^0=\textrm{cst},\; \forall i$.

Examples of numerical realizations of soliton condensates and diluted soliton condensates are given in Sects. 4.1, 6, 7 and in Appendix B. Figures 2, 4 and 20 show that numerical approximations of a condensate via the *n*-soliton solution are not exactly uniform; the realizations become more uniform near the center of the interval $[-\ell ,\ell ]$ as the number of soliton *n* increases.

The realization at $t=0$ in Figs. 7, 8, 10 and 12 also displays the “border effects” observed at the discontinuities of the Riemann problem initial condition (located at $x=0$). These border effects, manifesting as overshoots of the realization, persist regardless of the number of solitons *n* as shown by the comparison between the 100-soliton and 200-soliton solutions in Fig. 19. However, because of their finite size, the observed border effects seem to have no effect on the asymptotic dynamics of the condensate as demonstrated by the very good agreement between the theory and the numerical solution in Sect. 6.

### Generation of Spectral Parameters $\eta _i$

The spectral parameters of the *n*-soliton solution are distributed with probability density $\phi (\eta )$, cf. (A.3). This can be achieved by choosing the solutions of the nonlinear equation

A.8 $$\begin{aligned} \int _0^{\eta _i} \phi (\mu ) \textrm{d}\mu = \frac{i}{n},\quad i=1, \dots , n. \end{aligned}$$

For genus 0 condensate whose DOS is given by (4.1), this equation reduces to

A.9 $$\begin{aligned} 1-\sqrt{1-\frac{\eta _i^2}{\lambda _1^2}}= \frac{i}{n}. \end{aligned}$$

For genus 1 condensate with DOS (4.6), this equation reads

A.10 $$\begin{aligned} \frac{U(\eta _i)}{U(\lambda _3)} = \frac{i}{n},\quad \text {where:}\quad U(\eta ) = \int _0^\eta u^{(1)}(\mu ;\lambda _1,\lambda _2,\lambda _3) \textrm{d}\mu . \end{aligned}$$

We have

A.11 $$\begin{aligned} U(\eta ) = \int _0^\eta \frac{i(\mu ^2-w^2)}{2\pi \sqrt{R(\mu )}} \textrm{d}(\mu ^2) = \int _0^{\eta ^2} \frac{i(x-w^2)}{2\pi \sqrt{(x-\lambda _1^2)(x-\lambda _2^2)(x-\lambda _3^2)}} \textrm{d}x,\qquad \end{aligned}$$

which yields

A.12 $$\begin{aligned}{} & {} U(\eta ) = U_0 \nonumber \\{} & {} + {\left\{ \begin{array}{ll} \displaystyle - \frac{1}{\pi } \left[ \sqrt{\frac{(\lambda _3^2-\eta ^2)(\lambda _1^2-\eta ^2)}{\lambda _2^2-\eta ^2}} - \frac{\lambda _1^2-w^2}{\sqrt{\lambda _3^2-\lambda _1^2}}F(\beta ,q)- \sqrt{\lambda _3^2-\lambda _1^2}E(\beta ,q) \right] , &{} 0<\eta<\lambda _1, \\ 0, &{} \lambda _1<\eta<\lambda _2, \\ \displaystyle \frac{1}{\pi } \left[ \frac{\lambda _1^2-w^2}{\sqrt{\lambda _3^2-\lambda _1^2}}F(\kappa ,q) + \frac{\lambda _2^2-\lambda _1^2}{\sqrt{\lambda _3^2-\lambda _1^2}}\Pi (q,\kappa ,q) \right] , &{} \lambda _2< \eta <\lambda _3, \end{array}\right. }\nonumber \\ \end{aligned}$$

where

A.13 $$\begin{aligned}&\beta = \sin ^{-1} \left( \sqrt{\frac{\lambda _1^2-\eta ^2}{\lambda _2^2-\eta ^2}} \right) , \beta _0 = \sin ^{-1} \left( \frac{\lambda _1}{\lambda _2} \right) , \end{aligned}$$

A.14 $$\begin{aligned}&\kappa =\sin ^{-1} \left( \sqrt{\frac{(\lambda _3^2-\lambda _1^2)(\eta ^2-\lambda _2^2)}{(\lambda _3^2-\lambda _2^2)(\eta ^2-\lambda _1^2)}} \right) , \end{aligned}$$

A.15 $$\begin{aligned}&q = \frac{\lambda _3^2-\lambda _2^2}{\lambda _3^2-\lambda _1^2}, \end{aligned}$$

A.16 $$\begin{aligned}&\begin{aligned}&U_0 \!=\! \frac{1}{\pi } \left[ \sqrt{\frac{\lambda _3^2\lambda _1^2}{\lambda _2^2}} - \frac{\lambda _1^2-w^2}{ \sqrt{\lambda _3^2\!-\!\lambda _1^2}}F(\beta _0,q)\!-\!\sqrt{\lambda _3^2-\lambda _1^2}E(\beta _0,q) \right] . \end{aligned} \end{aligned}$$

### Algorithm for the *n*-soliton Solution

The algorithm generating the exact *n*-soliton (A.1), originally developed in Huang (1992), relies on the Darboux transformation. This scheme is subject to roundoff errors during summation of exponentially small and large values for a large number of solitons *n*. We improve it following Gelash and Agafontsev (2018), with the implementation of high-precision arithmetic routine to overcome the numerical accuracy problems and generate solutions with a number of solitons $n \gtrsim 10$.

In order to simplify the algorithm, it is suggested to consider simultaneously the KdV equation (2.1) and its equivalent form

A.17 $$\begin{aligned} \varphi _t-6\varphi \varphi _x+\varphi _{xxx}=0 \end{aligned}$$

obtained from (2.1) by the reflection $\varphi \rightarrow - \varphi$. The Darboux transformation presented here relates the Jost solution associated with the $(n-1)$-soliton solution of one equation, to the *n*-soliton solution of the other equation.

Considering the direct scattering problem for the Lax pair in the matrix form

A.18 $$\begin{aligned} \Phi _{x}=\left( \begin{array}{cc} \eta &{} {\mp } 1 \\ \varphi &{} - \eta \end{array}\right) \Phi , \end{aligned}$$

with $-1$ corresponding to (2.1) and $+1$ to (A.17), the Jost solutions $J,\tilde{J}\in {\mathbb {R}}^{2 \times 2}$ are defined recursively by the Darboux transformations $D(\eta )$ and $\tilde{D}(\eta )$ such that:

A.19 $$\begin{aligned} J_{n}(\eta )= & {} D_{n}(\eta ) J_{n-1}(\eta ),\quad \text {with:} \quad D_{n}(\eta )=I+\frac{2\eta _{n}}{\eta -\eta _{n}} P_{n}, \end{aligned}$$

A.20 $$\begin{aligned} \tilde{J}_{n}(\eta )= & {} \tilde{D}_{n}(\eta ) \tilde{J}_{n-1}(\eta ), \quad \text {with:} \quad \tilde{D}_{n}(\eta )=I-\frac{2\eta _{n}}{\eta +\eta _{n}} \tilde{P}_{n}. \end{aligned}$$

$P_n(x,t)$ and $\tilde{P}_n(x,t)$ are independent of $\eta$ and have the form:

A.21 $$\begin{aligned} P_{n}=\sigma _2\tilde{P}^{ \text {T}}_{n}\sigma _2=\frac{J_{n-1}\left( -\eta _{n}\right) \left( \begin{array}{c} -b_n \\ 1 \end{array}\right) \left( \begin{array}{ll} b_n&1 \end{array}\right) \tilde{J}_{n-1}^{-1}\left( \eta _{n}\right) }{\left( \begin{array}{ll} b_n&1 \end{array}\right) \tilde{J}_{n-1}^{-1}\left( \eta _{n}\right) J_{n-1}\left( -\eta _{n}\right) \left( \begin{array}{c} -b_n \\ 1 \end{array}\right) }, \end{aligned}$$

with the real constants $b_n$ depending on the spatial phases

A.22 $$\begin{aligned} b_n=\left( -1\right) ^{n}\exp \left( 2\eta _n x^{0}_{n}\right) . \end{aligned}$$

The Jost solutions for the initial seed solution $\varphi _0=0$ are given by:

A.23 $$\begin{aligned} J_{0}(\eta )=\tilde{J}_{0}(\eta )=\left( \begin{array}{cc} \exp \left[ \eta x-4 \eta ^{3} t\right] &{} -\exp \left[ -\eta x+4 \eta ^{3} t\right] \\ 0 &{} - 2 \eta \exp \left[ -\eta x+4 \eta ^{3} t\right] \end{array}\right) , \end{aligned}$$

and one can show that at each recursion step

A.24 $$\begin{aligned} \varphi _{n}=\varphi _{n-1}+4\eta _{n}\left( P_{n}\right) _{21}, \end{aligned}$$

where $\varphi _n$ is the *n*-soliton solution of (2.1) for *n* even and solution of (A.17) for *n* odd. Recently, a more efficient and accurate algorithm has been proposed in Prins and Wahls (2021) to generate the *n*-soliton KdV solution employing a 2-fold Crum transform.

## Genus 2 Condensate

In Fig. 20, a realization of the genus 2 soliton condensate is compared with the two-phase KdV solution associated with the same spectral surface and equipped with an appropriately chosen initial phase vector. The two-phase solution has been computed numerically using the so-called trace formula (Novikov et al. 1984):

B.1 $$\begin{aligned} \varphi (x, t)=\lambda _{1}^2-2 \sum _{j=1}^{2}\left( \mu _{j}^2(x, t)-\frac{\lambda _{2 j}^2+\lambda _{2 j+1}^2}{2}\right) , \end{aligned}$$

where   the   auxiliary spectra $\mu _j(x,t)$ satisfy Dubrovin’s ordinary differential equations:

B.2 $$\begin{aligned} \begin{aligned} \frac{\partial (\mu _j^2)}{\partial x}=\frac{2 \sigma _{j} R \left( \mu _{j}\right) }{\prod _{j \ne k}^{2}\left( \mu _{j}^2-\mu _{k}^2\right) }, \quad j=1,2, \end{aligned} \end{aligned}$$

with $$\sigma =\pm 1$$ and

B.3 $$\begin{aligned} R(\mu )=\sqrt{ \left( \mu ^2-\lambda _{1}^2\right) \left( \mu ^2-\lambda _{2}^2\right) \left( \mu ^2-\lambda _{3}^2\right) \left( \mu ^2-\lambda _{4}^2\right) \left( \mu ^2-\lambda _{5}^2\right) }. \end{aligned}$$

Each $\mu _j$ oscillates in the corresponding s-gap $[\lambda _{2j-1},\lambda _{2j}]$ so that the sign of $\sigma _j$ changes every time $\mu _j$ changes the direction of motion at the gap end point. We observe that, to compute the KdV finite-gap solution corresponding to a given realization soliton condensate, all the initial phases $\mu _j(x_0,t)$ must be placed at the edges of the corresponding s-gaps, while the choice of the gap’s edge (right/left) is determined by the number of discrete eigenvalues (odd/even) that are located within the s-band $[\lambda _{2j},\lambda _{2j+1}]$ in the numerical construction of the condensate.
